# Zinc–Acetate–Amine Complexes as Precursors to ZnO and the Effect of the Amine on Nanoparticle Morphology, Size, and Photocatalytic Activity

**DOI:** 10.3390/catal12101099

**Published:** 2022-09-23

**Authors:** Jerry D. Harris, Emily A. Wade, Emmaline G. Ellison, Cecelia C. Pena, Stephen C. Bryant, Nicholas L. McKibben, Allison J. Christy, Kevin O. Laughlin, Ashley E. Harris, Kenrik V. Goettsche, Chad E. Larson, Seth M. Hubbard, Jonathan E. Cowen, Josh Eixenberger, David Estrada, Jennifer R. Chase

**Affiliations:** 1 Department of Chemistry, Northwest Nazarene University, Nampa, ID 83686, USA; 2 Department of Physics, Rochester Institute of Technology, Rochester, NY 14623, USA; 3 Swagelok Center for the Surface Analysis, Case Western Reserve University, Cleveland, OH 44106, USA; 4 Micron School of Materials Science and Engineering, Boise State University, Boise, ID 83725, USA; 5 Department of Biology, Northwest Nazarene University, Nampa, ID 83686, USA

**Keywords:** nanoparticles, photocatalyst, thermal analysis, mass spectrometry, surface area, synthesis, alkali precipitation, diffuse reflectance

## Abstract

Zinc oxide is an environmentally friendly and readily synthesized semiconductor with many industrial applications. ZnO powders were prepared by alkali precipitation using different [Zn(acetate)_2_(amine)_x_] compounds to alter the particle size and aspect ratio. Slow precipitations from 95 °C solutions produced micron-scale particles with morphologies of hexagonal plates, rods, and needles, depending on the precursor used. Powders prepared at 65 °C with rapid precipitation yielded particles with minimal morphology differences, but particle size was dependent on the precursor used. The smallest particles were produced using precursors that yielded crystals with low aspect ratios during high-temperature synthesis. Particles produced during rapid synthesis had sizes ranging from 21–45 nm. The materials were characterized by scanning electron microscopy, transmission electron microscopy, X-ray diffraction, thermogravimetric analysis, BET, and diffuse reflectance. The materials prepared using precursors with less-volatile amines were found to retain more organic material than ZnO produced using precursors with more volatile amines. The amount of organic material associated with the nanoparticles influenced the photocatalytic activity of the ZnO, with powders containing less organic material producing faster rate constants for the decolorizing of malachite green solutions under ultraviolet illumination, independent of particle size. [Zn(acetate)_2_(hydrazine)_2_] produced ZnO with the fastest rate constant and was recycled five times for dye degradation studies that revealed minimal to no reduction in catalytic efficiency.

## 1. Introduction

Zinc oxide is a II-VI-wide bandgap semiconductor that has many industrial applications [1]. It is relatively inexpensive, easy to prepare, has attractive electronic properties, and is an environmentally friendly alternative to other semiconductors, such as CdS and ZnSe [2]. Like other materials, the physicochemical properties of ZnO nanoparticles show various enhancements compared to bulk ZnO. These enhancements include, but are not limited to, increased optical properties and sensor sensitivity [3,4], increased reactivity toward cancer cells and bacteria [5,6], and increased catalytic activity [7]. Both particle size and shape influence these enhancements. Micro- and nanoparticles of ZnO have been prepared in a wide range of shapes and sizes that have included nanorods [8,9], nanowires [8,10], nanotubes [11,12], nanosheets [13,14], plates [15,16], and multi-segmented structures [17-19]. The richness of the size and morphologies obtained is dependent on the synthesis method (sol-gel, precipitation, hydrothermal, etc.), other molecules and ions present during synthesis [20-23], solvents used [15,24], rate of precipitation [19], and annealing time and temperature [25,26].

Air-stable molecules used to prepare ZnO include zinc acetate, zinc chloride, zinc nitrate, and zinc acetylacetonate [3,27-35]. All of these compounds are water-soluble, enabling a greener synthesis, and their physicochemical properties can be modified by adding other ligands. Amines as ligands have been utilized during ZnO synthesis to act as solution stabilizers, modify decomposition temperatures and solubility, used as N-doping sources, and used as capping ligands on nanoparticles, but the precursor molecules are seldom isolated and characterized [29,31,36-38].

In our previous work, we isolated, characterized and utilized [Zn(acetate)_2_(amine)_2_] single-source precursors to prepare ZnO films and powders [39]. We demonstrated that the size and morphology of ZnO particles prepared by alkali precipitation could be modified using different amines and changing the precipitation rate. However, several precursors lost solubility during the work, upon isolation and subsequent storage under ambient conditions and in an inert nitrogen-filled glove glovebox. For this work, to minimize solubility issues, the [Zn(acetate)_2_(amine)_x_] precursors were generated in solution, and the ZnO nanoparticles were prepared by alkali precipitation without the additional steps of isolation and redissolution. Maintaining the precursors in solution simplifies the synthesis of ZnO, decreases the synthesis time, and allows more amines to be used in the synthesis while still using fully characterized precursors.

The synthesis and characterization of several of the materials made using [Zn(acetate)_2_(amine)_x_] compounds are presented here. The presence of the different amines influences the physicochemical properties (particle size and shape, surface area, etc.) of the precipitated particles. To explore how modifying these properties affects ZnO’s photocatalytic properties, the nanomaterials were used to photocatalytically decolorize solutions of the triarylmethane dye malachite green. Malachite green is used in many industrial applications, including dyeing textiles, plastics, and paper. It is also used as a fungicide and parasiticide in aquaculture applications, but its use in many countries has been banned because of its toxicity to animals and humans [40,41]. Therefore, it is desirable to develop low-cost, effective processes for removing malachite green from industrial effluents and the environment using eco-friendly chemicals such as ZnO nanoparticles. Many have studied ZnO as a potential photocatalyst for water treatment [42,43]. Here, we demonstrate how the physicochemical properties of ZnO nanoparticles can be modified using [Zn(acetate)_2_(amine)_x_] precursors and how these modifications impact the material’s ability to degrade an organic dye. Using these materials with the simplified synthetic process may provide an effective solution to treating wastewater. Additionally, ZnO composites with chitosan, graphene, graphene oxide, and carbon nanotubes have recently demonstrated excellent photocatalytic activity for degrading organic dyes [44-48]. One of the benefits of using ZnO composites as the photocatalyst is that they minimize the photocorrosion that can occur to ZnO in solution.

However, few authors who report ZnO photocatalysis ever characterize their ZnO precursors. Many use simple Zn^2+^ salts as their zinc source, while others use zinc-containing molecules or zinc ions with other ions in solution, but seldom do they report the characterization of their zinc oxide precursors [44-46,49-60]. In contrast, here we report the synthesis, NMR characterization, and CHN elemental analysis of the precursors. We have previously reported the crystal structure of [Zn(acetate)(ethylenediamine)] [39]. Characterizing the precursors allows for the correlation of precursor composition with the ZnO particle size and shape, the kind and mount of organic ligands associated with the ZnO, and the material’s photocatalytic performance. Additionally, the materials reported here have similar photocatalytic performance to the ZnO composite materials.

## 2. Results and Discussion

### 2.1. Materials Characterization

Several [Zn(acetate)_2_(amine)_x_] compounds were synthesized and characterized. The compounds were isolated from solution and characterized by carbon, hydrogen, and nitrogen elemental analysis and NMR to verify that the precursors had the correct stoichiometry. For the amines utilized extensively in this work (Tris, 2-thiazolamine, hydrazine, and ethylenediamine), the analysis results are consistent with tetrahedrally coordinated zinc compounds, with both the amines and acetates binding at two coordination sites around the zinc. Tris, 2-thiazolamine, and hydrazine yield compounds that contain two amines bound to the zinc, whereas the compound prepared using ethylenediamine has only one of the bidentate ligands bound to the zinc. All precursor compounds are soluble and were characterized by NMR, except the one prepared with hydrazine. The NMR experiments and elemental analysis are consistent with the expected stoichiometry. The crystal structure of [Zn(acetate)_2_(ethylenediamine)] was reported in our previous work [39].

The compounds were maintained in solution and then used to prepare ZnO nanoparticles by alkali precipitation. For this method, a zinc ion in solution is precipitated by adding an alkali hydroxide. The precipitate is then isolated, washed, dried, and annealed or calcined to convert any Zn(OH)_2_ to ZnO and remove any solvent still associated with the product [61]. Zinc is an amphoteric element and, without coordinating ligands, can precipitate as Zn(OH)_2_ at pHs near neutral. At higher pHs, ZnO can be formed from hydrolysis and condensation, leading to quasi-spherical particles if there is no preferential growth along the c-axis [62,63]. Mechanisms for the growth of ZnO nanoparticles from Zn(acetate)_2_ in the presence of amines have been proposed by several investigators, and all utilize Zn(OH)_2_ and Zn_4_O(acetate)_6_ intermediates [38,64,65].

**Figure 1.**
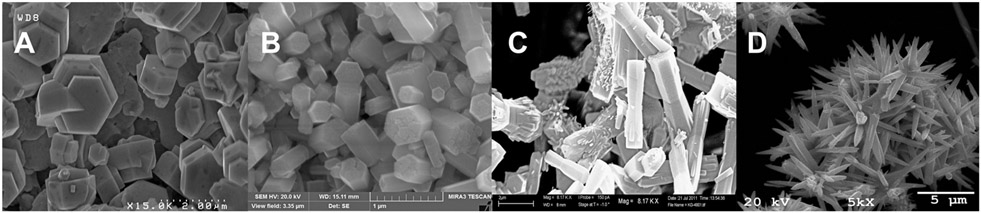
SEM images of ZnO powders prepared from [Zn(acetate)_2_(amine)_2_] precursors at 95°C in an aqueous solution. The precursors used were (**A**) [Zn(acetate)_2_(Tris)_2_], (**B**) [Zn(acetate)_2_(2-thiazolamine)_2_], (**C**) [Zn(acetate)_2_(hydrazine)_2_], and (**D**) [Zn(acetate)_2_(ethylenediamine)].

Ligands in solution during particle growth and precipitation can be used to modify the shape and size of the ZnO particles. Meagley and Garcia modified the aspect ratio of ZnO particles using carboxylate ligands of different denticity [22]. In their work, no added ligands and monodentate ligands yielded long rods, bidentate ligands yielded short rods, and tridentate ligands yielded plates. The [Zn(acetate)_2_(amine)_x_] molecules in this work do not follow this trend during alkali precipitation. Hydrazine and 2-thiazolamine yield rods, ethylenediamine yields long needles, and Tris yields plates when grown at 95°C in aqueous solutions (Figure 1). Particle sizes are 700 nm × 1 μm hexagonal plates for Tris, 700 nm × 200 nm rods for 2-thiazolamine, 8 μm × 2 μm rods for hydrazine, and 5 μm × 500 nm needles for ethylenediamine when prepared in water and with the slow addition of NaOH over 30 min. If zinc acetate is used without any added amine, round rods are formed. When using [Zn(cyclohexyl)_2_(hexadecylamine)] to prepare ZnO, Zheng et al. found temperature to be the primary factor controlling morphology, with spherical nanoparticles produced at temperatures over 60°C and rods produced at temperatures less than 60 °C [66]. Zhang et al. used zinc acetate solutions containing different long-chained amines (oleylamine, dioctylamine, hexadecylamine, and dodecylamine) to modify particle morphology and produced ZnO nanoparticles with shapes ranging from prisms to wires to rods [29]. Mechanisms for the growth of nanoparticles from precursors have been proposed but have been dependent on the synthetic protocol and required optimization for a particular morphology from a specific precursor [67].

**Figure 2.**
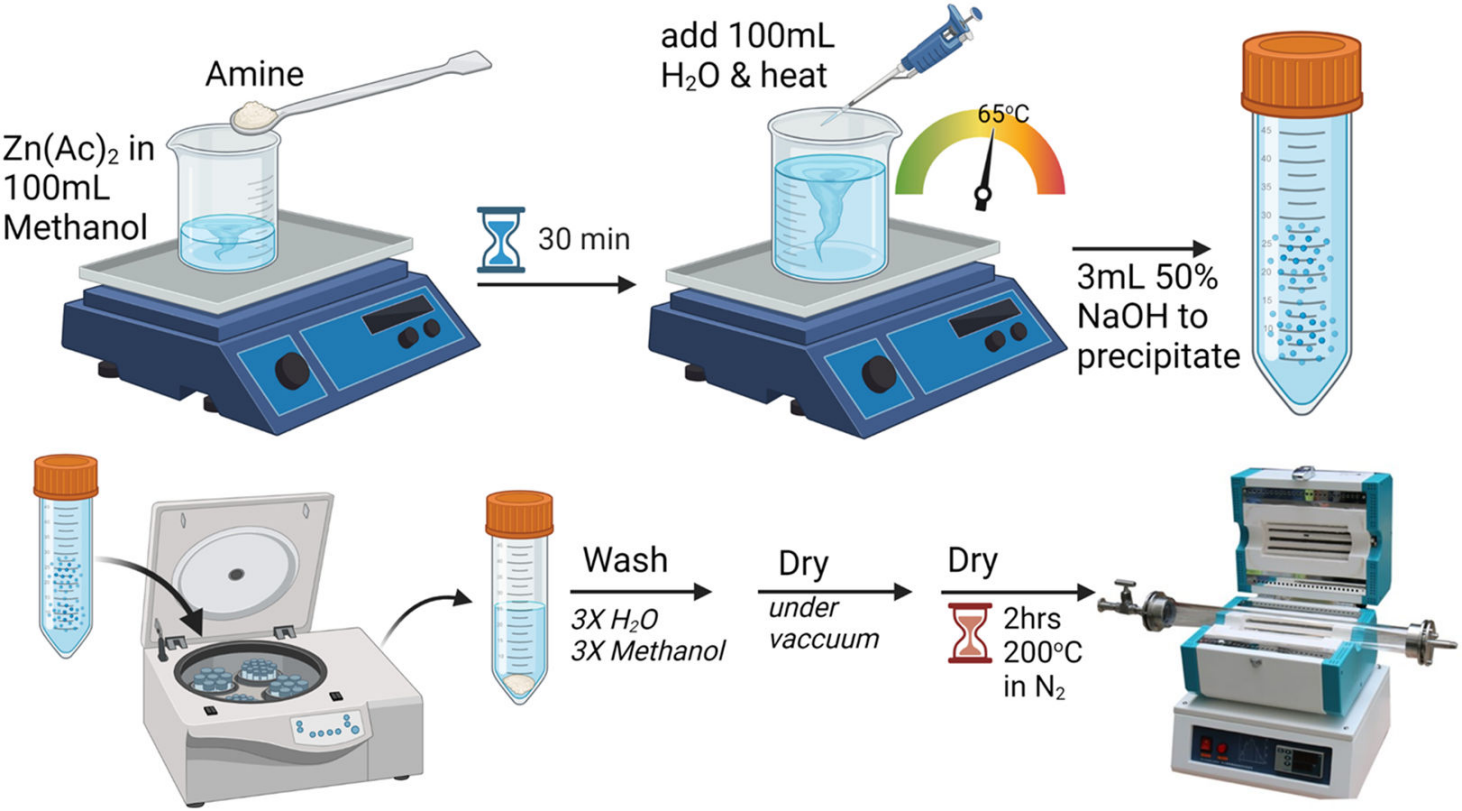
A schematic for the synthesis of ZnO from [Zn(acetate)_2_(amine)_x_] precursors with rapid addition of NaOH to precipitate the product. This figure was created with BioRender.com.

**Figure 3.**
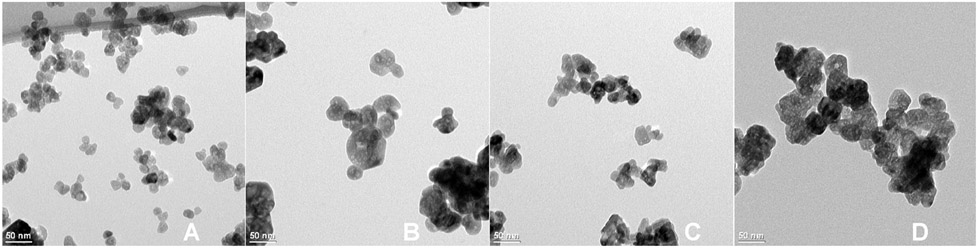
TEM images of ZnO nanoparticles prepared from [Zn(acetate)_2_(amine)_2_] precursors in a water/methanol mixture with rapid NaOH addition at 65 °C. Average particle size are given for each materaial. The precursors used were (**A**) [Zn(acetate)_2_(Tris)_2_] (20 ± 4 nm), (**B**) [Zn(acetate)_2_(2-thiazolamine)_2_] (34 ± 9 nm), (**C**) [Zn(acetate)_2_(hydrazine)_2_] (27 ± 7 nm), and (**D**) [Zn(acetate)_2_(ethylenediamine)] (45 ± 7 nm).

Since particle size is likely driven by nucleation kinetics, lower temperatures and faster precipitation rates were expected to yield smaller particles because of the limited nucleation time during rapid precipitation. To achieve this, the solvent was changed from 100% water to a 50/50 mixture of methanol/water to prevent early precipitation before NaOH addition, the synthesis temperature was lowered to 65 °C, and the NaOH was added rapidly. A schematic of the rapid synthesis protocol is provided in Figure 2. For these experiments, we utilized the same amine precursors that produced micron-sized particles and the various morphologies seen in Figure 1, (Tris = plates, 2-thiazolamine = short rods, hydrazine = long rods, and ethylenediamine = long needles). Under these synthesis conditions, the size of the particles decreases to 20–45 nm, depending on which precursor was used. Unlike the previous high temperature, slow NaOH addition route, the morphology differences were minimal with the low temperature, rapid NaOH addition route (Figure 3). Close inspection reveals hexagonal morphology is still observed in the nanoparticles. In addition to temperature and precipitation rate, the amine on the precursor also influenced the particle size. Precursors that produce large decreases in the particle aspect ratio in the slow precipitation method also cause the greatest size reduction during rapid precipitation. For example, [Zn(acetate)_2_(Tris)_2_] yielded plates with a 0.7 to 1 (L to W) aspect ratio with slow precipitation when prepared at 95 °C and under the rapid precipitation protocol yielded the smallest particles (20 nm). [Zn(acetate)_2_(ethylenediamine)] produced crystals with a 10 to 1 aspect ratio during slow precipitation at 95 °C and during rapid precipitation yielded the largest particles (45 nm). [Zn(acetate)_2_(2-thiazolamine)_2_] and [Zn(acetate)_2_(hydrazine)_2_] produced particles with intermediate aspect ratios of 3.1 to 1 and 4 to 1, respectively, during slow precipitation at 95 °C and yielded comparably sized particles during the rapid synthesis (34 nm and 27 nm, respectively).

**Figure 4.**
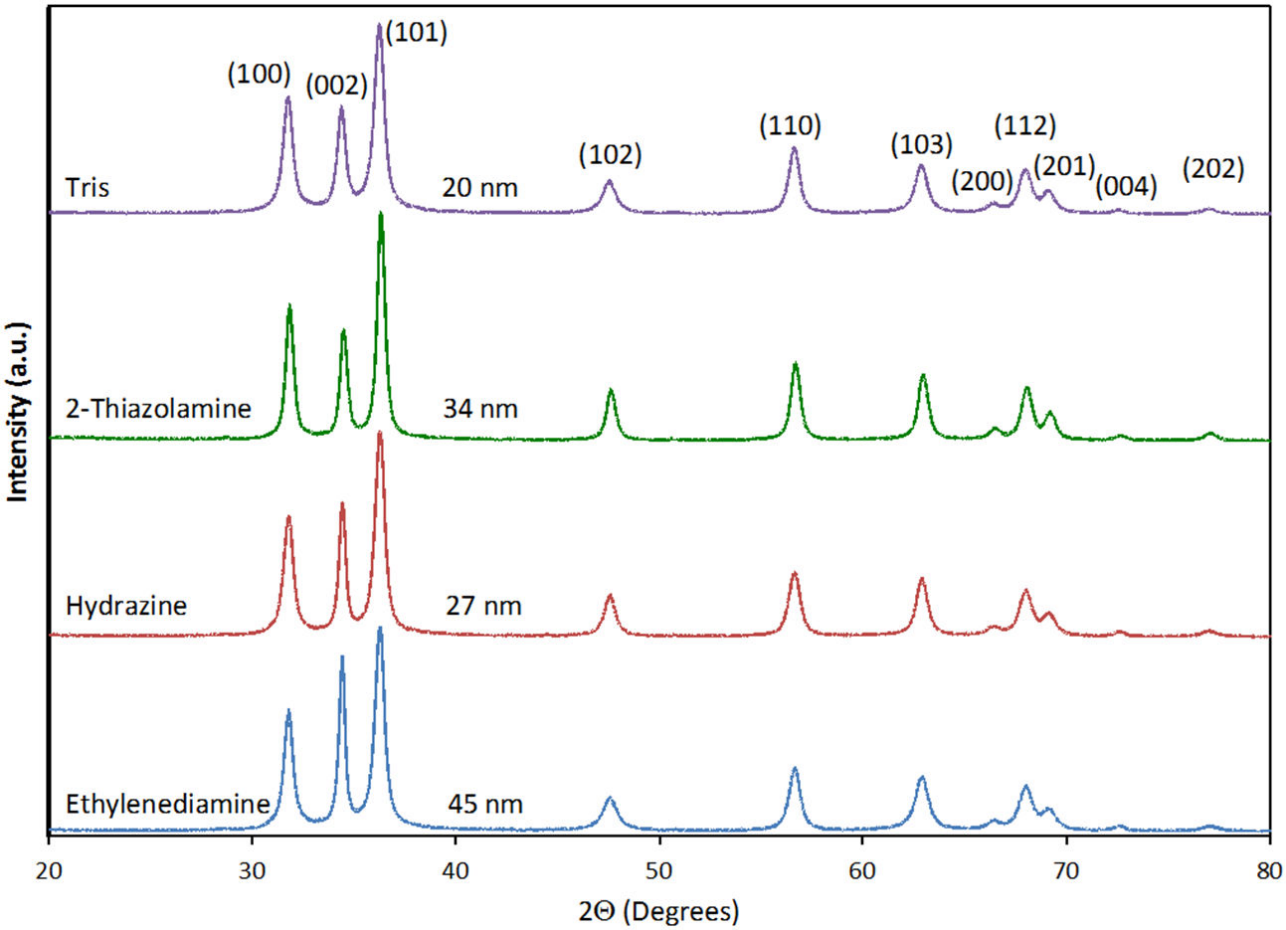
X-ray powder diffraction data for ZnO nanomaterials prepared using [Zn(acetate)_2_(amine)_2_] precursors by alkali precipitation in water/methanol solutions at 65°C. The labels are the amine on the precursor during synthesis. The average particle size given is that observed with the TEM measurements.

All ZnO powders were found to be phase pure wurtzite and highly crystalline when characterized by X-ray powder diffraction (Figure 4). The average particle size was also calculated using the Scherrer equation and diffraction data from the (100), (002), and (101) peaks. Calculated particle sizes ranged from 15–19 nm (Table 1). The Scherrer equation calculations indicate that the particles observed in the TEM are aggregates of small crystallites. The particle sizes are consistent with what others have observed for precursor grown ZnO prepared by precipitation and sol-gel synthesis, with particle sizes ranging from 10–165 nm [63,68].

The surface area of the nanoparticles was obtained using the BET method from nitrogen adsorption/desorption isotherms to determine how the different precursors used during synthesis influence the surface area of the materials (Table 1). As expected, ZnO with smaller particle sizes produced higher surface areas. Surface areas ranged from 16.1 m^2^/g for 45 nm ZnO particles produced using ethylenediamine to 43.7 m^2^/g for 20 nm ZnO particles prepared using Tris. These surface areas are consistent with results obtained by others [68,69]. Absorption/desorption isotherms for each compound are provided in the supplemental material (Figures S1-S4). Particle size and surface area are related by Equation (1), allowing the surface area results to be used as another means of determining particle size [70]. The equation assumes that all particles are solid spheres of uniform size with a smooth surface, which is not strictly true given the hexagonal shape of these nanoparticles. However, the equation does provide another means of determining the particle size for each material. The average particle size, *D*_*BET*_ (nm), is determined using the theoretical density of the material, *ρ*, (ZnO = 5.61 g/cm^3^), and the specific surface area obtained from the BET measurements, *S*_*BET*_ (m^2^/g). Equation 1 calculates particle sizes of 24.5 nm for the nanoparticles made using the precursor [Zn(acetate)_2_(Tris)_2_], 30.5 nm with that prepared using [Zn(acetate)_2_(2-thiazolamine)_2_], 30.0 nm with [Zn(acetate)_2_(hydrazine)_2_], and 66.4 nm for the particles made using [Zn(acetate)_2_(ethylenediamine)]. These calculated values are consistent with the particle sizes observed with TEM measurements.


(1) DBET=∕ρ⋅SBET6000

**Figure 5.**
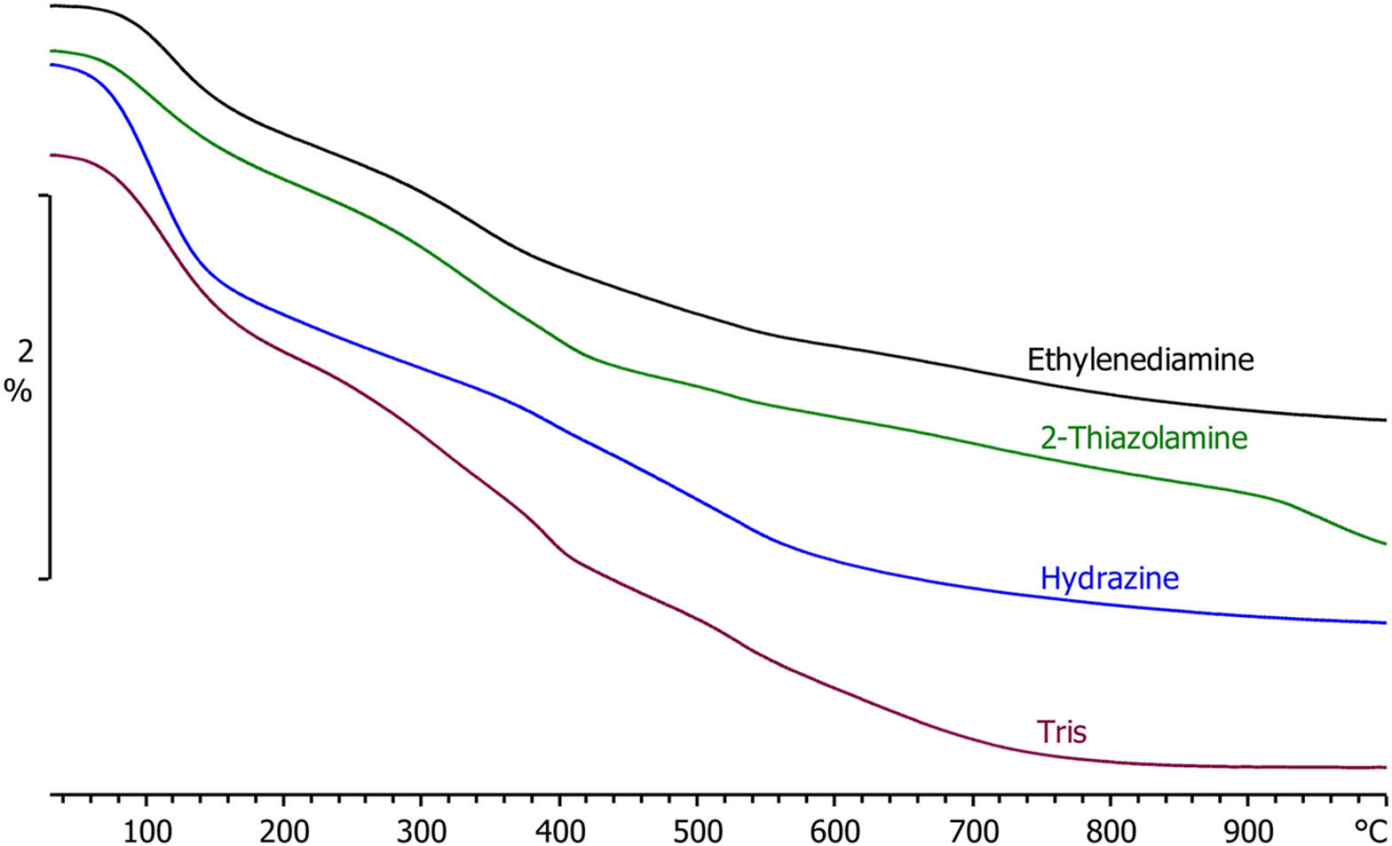
TGA curves showing the percent weight loss during heating for ZnO powders prepared using four different precursors. The amine label by the curve corresponds to the amine used during synthesis of the ZnO.

Thermogravimetric analysis—mass spectrometry (TGA-MS) was also used to characterize the materials prepared by rapid precipitation. The TGA data revealed that ZnO prepared with lower boiling amines resulted in less mass loss when heated to 1000 °C (Figure 5). Since all the materials were annealed at 200 °C as part of the synthesis, the observed weight loss under 200 °C in the TGA was assumed to be from loss of adsorbed atmospheric moisture. All of the materials were observed to have some degree of hygroscopicity when removed from the annealing furnace and weighed for yield. The mass loss above 200 °C was assumed to be from decomposition and volatilization of capping ligands (acetate and amines) attached to the zinc oxide nanoparticles. The materials prepared using Tris, 2-thiazolamine, hydrazine, and ethylenediamine lose 2.2%, 1.8%, 1.6%, and 1.5% of their mass, respectively, when heated from 200 °C to 1000 °C in the TGA. Hydrazine and ethylenediamine have boiling points less than the 200 °C annealing temperature (114 °C and 116 °C, respectively), whereas 2-thiazolamine and Tris have boiling points higher than the annealing temperature (216 °C and 219 °C, respectively). Consistent with the material retaining some of the amines, ZnO prepared using Tris and 2-thiazolamine lose more mass above 200 °C when heated to 1000 °C than hydrazine and ethylenediamine.

To better understand the thermal decomposition results, to confirm the identity of the materials lost during heating, and to help quantify the number and kinds of ligands attached to the nanoparticles, all materials were characterized by TGA-MS. Additionally, nanoparticles were made using Zn(NO_3_)_2_ with the added amines and Zn(acetate)_2_ with no added amines and were characterized by TGA-MS to help understand how the reactants affect the final materials. Individual TGA-MS data for each ZnO material are provided in the supplemental material (Figures S5-S13). For these nanomaterials, the majority of the mass loss below 200 °C comes from the loss of H_2_O, which starts to be released at 30–50 °C, has a maximum around 100 °C, and trails off by 200 °C. Additionally, all materials made using amines, except 2-thiazolamine, release CO_2_ concurrent with the low temperature release of H_2_O. Materials prepared using Zn(acetate)_2_ and hydrazine or Tris also release NO_2_ at the same time as their low temperature release of H_2_O and CO_2_. These results are consistent with ZnO previously being shown to absorb CO_2_ and NO_2_ and ZnO’s use in sensors for CO_2_ and NO_2_ detection [71,72].

To help determine how much acetate is still attached to each nanomaterial, ZnO was prepared using Zn(acetate)_2_ with no added amine. The TGA data for this material show a pronounced weight loss starting around 250 °C, from the loss of water, carbon dioxide, and the acetate ligand (m/z = 58) (Figure S9). The m/z = 58 was monitored because that mass to charge ratio was found to have the highest intensity during the TGA-MS characterization of solid Zn(acetate)_2_. The acetate signal peaks at 300 °C and is gone by 360 °C. However, the signals for water and CO_2_ continue to increase until about 400 °C, indicating that the remaining acetate ligands start to be converted to CO_2_ and water above 300 °C and are entirely burned above 360 °C. All remaining hydrocarbons are converted to CO_2_ and H_2_O as the temperature ramps to 1000 °C, yielding a 2.7% mass loss during the heating from 200 °C to 1000 °C.

The TGA-MS data for zinc oxide prepared using zinc acetate with no added amines provide insight into the composition of the nanoparticles prepared using Zn(acetate)_2_ with added amines. No acetate signal is observed in the mass spectrometer for any samples prepared using zinc acetate and amines, indicating that minimal acetate is still attached to the material. It has been proposed that the amine can nucleophilic attack the acetate carbonyl and cause the elimination of the amide during zinc oxide synthesis [29]. This would explain why acetate ions are not retained in some of the materials. The lack of acetate on these materials gives them a lower mass loss than the ZnO made from Zn(acetate)_2_ and no amines. To help confirm the lack of acetate ions, ZnO nanomaterials were also prepared using zinc nitrate and each amine, and the materials were analyzed by TGA-MS (Figures S10-S13). The ZnO samples made using ethylenediamine and both zinc sources have similar weight losses (to two significant digits) from 200 °C to 1000 °C. Although the sample prepared using Zn(acetate)_2_ has a higher CO_2_ loss above 600 °C than the sample using zinc nitrate, this sample likely contains minimal acetate since the acetate signal is missing in the MS. Neither of these samples had a mass spectrometer signal for NO or NO_2_, or any other mass associated with loss of the diamine, indicating that it was lost either during precipitation or the 200 °C annealing step.

In comparing the TGA-MS data for the materials prepared using hydrazine and the two zinc sources, the sample prepared using the nitrate had a larger mass loss than the sample prepared using zinc acetate, with much of the mass lost as water between 200 °C and 600 °C. Since this is well above the vaporization temperature of water, this is likely from hydroxides on the particle surface decomposing to water and forming the surface oxide [61,73]. The sample prepared using zinc acetate does show a small CO_2_ loss with a peak near 390 °C, but likely contains only minimal acetate ions, given the lack of observed acetate signal in the MS. These samples also lose tiny amounts of NO and NO_2_ at the same temperature as the CO_2_ loss, indicating that most of the hydrazine was lost during the synthesis or annealing steps.

Zinc oxide samples made using zinc acetate and the two less volatile amines show minimal low temperature (<200 °C) loss of CO_2_ in the TGA-MS, indicating that the ZnO surface of these particles might be partially covered with amines or acetate ligands and not readily accessible for absorbing CO_2_. In the samples made using zinc acetate and Tris, CO_2_ and NO_2_ start to be lost a little above 200 °C, and the shape of the CO_2_ signal resembles that of the CO_2_ signal for the zinc oxide prepared using zinc acetate with no added amines. Both of the samples made with Tris continue to produce CO_2_ and H_2_O to at least 800 °C, consistent with them burning off attached hydrocarbons. The sample made using Tris and zinc acetate loses 2.2% total mass during heating from 200 °C to 1000 °C compared to the sample made with zinc nitrate losing 1.9% mass during the same temperature interval. This greater weight loss is consistent with the sample made using zinc acetate retaining some of the acetate on the particles.

The materials prepared using 2-thiazolamine produce similar TGA signals, with each having a high temperature mass loss which starts at 850 °C for the sample made with zinc nitrate and 920 °C for the material made using zinc acetate. The product made with zinc acetate also has a higher mass loss of 1.8%, compared to 1.7% for the sample made using zinc nitrate. The mass spectrometry data for the two products poorly resemble each other. For the product made with Zn(NO_3_)_2_, the signal from CO_2_ peaks at 310 °C, with a long trailing tail that plateaus from 380–410 °C, compared to the sample made using Zn(acetate)_2_, which peaks at 390 °C, dies away quickly and then climbs to a small peak at 920 °C. The data are consistent with both materials retaining some of the amine and continuing to burn it off all the way to 1000 °C. Additionally, the material made using zinc acetate likely retains some of the acetate ligands, given the small increase in mass loss over that of the sample made using zinc nitrate.

In an attempt to quantify the number of ligands associated with each ZnO prepared from zinc acetate, each material was characterized by CHN elemental analysis. However, only ZnO prepared using Tris and 2-thiazolamine had carbon percentages above the 0.5% reporting limit (Tris: C = 1.10% and 2-thiazolamine: C = 0.74%). Results for hydrogen and nitrogen were below the reporting limit for all samples, which could have been used to quantify the amount of amine bound to each ZnO. Using the carbon percentages obtained for the materials made from [Zn(acetate)_2_(Tris)_2_] and [Zn(acetate)_2_(2-thiazolamine)_2_], and assuming a composition of ZnO(acetate)_x_(amine)_x_ to determine stoichiometry, yields formulas of ZnO(acetate)_0.0128_(Tris)_0.0128_ (one set of ligands for every 78 ZnO formula units) and ZnO(acetate)_0.0103_(2-thiazolamine)_0.0103_ (one set of ligands for every 97 ZnO formula units). Assuming the observed mass loss in the TGA from 200–1000°C for these materials is from loss of attached ligands, the 2.2% mass loss for the material made using Tris would yield a calculated stoichiometry of ZnO(acetate)_0.0102_(Tris)_0.0102_. The 1.8% mass loss for 2-thiazolamine would yield ZnO(acetate)_0.0096_(2-thiazolamine)_0.0096_. These stoichiometries are in reasonably good agreement with the CHN data, considering both materials were still losing mass at the 1000 °C TGA cutoff temperature. Performing the same calculations for the material made with ethylenediamine and a 1.5% TGA mass loss would yield ZnO(acetate)_0.014_(ethylenediamine)_0.007_, based on the stoichiometry of the ligands on the precursor carrying over to the final composition of the ZnO. This stoichiometry is equivalent to a 0.6% carbon content and should have exceeded the elemental analysis detection limit. The fact that carbon was not detected in the CHN analysis supports the hypothesis that very little of the acetate was incorporated into the final material, or that most of the diamine was lost during the 200 °C annealing step, and that the extra weight loss observed in the TGA is from an undetermined number of hydroxides being lost as water during heating. Likewise, for the material prepared using hydrazine to have a 1.6% TGA weight loss would require a final stoichiometry of ZnO(acetate)_0.0146_(hydrazine)_0.0146_ and would correspond to a 0.4% carbon content and a 0.5% nitrogen content. Although the carbon content is below the CHN detection limit, the nitrogen content is at the detection limit. It could have been determined, further supporting the hypothesis that much of the weight loss for this material is from the loss of water from hydroxides. Attempts were also made to quantify the number of ligands bound to each ZnO using FT-IR and X-ray photoelectron spectroscopy. With such small quantities, vibrations associated with the functional groups on the ligands were not visible above the ZnO background in the IR spectra (Figure S14). Neither nitrogen nor sulfur was observed in the XPS spectrum for any of the materials, consistent with them containing a very limited number of ligands.

To better understand how the synthesis conditions influence the optical properties of the materials, the optical bandgap for each ZnO nanomaterial prepared by the rapid precipitation method was determined by ultraviolet-visible diffuse reflectance spectroscopy. The data were transformed using the Kubelka–Munk equation (Equation (2)) [74,75], where *R*_∞_ = *R*_sample_/*R*_standard_ and is the measured diffuse reflectance of the sample, *F* (*R*_∞_) is the Kubelka–Munk function or the so-called remission, *hν* is the photon energy, *C*_2_ is a proportionality constant, and *E*_*g*_ is the optical band gap of the powdered sample. The band gap is obtained from a plot of [*F* (*R*_∞_) *hν*]^2^ against *hν*, where a linear fit through the steepest part of the data intersects the photon energy axis (*x*-axis) is taken as the value for *E*_*g*_.


(2) $$\left[F\;(R_{\infty })\;hv{]}^{2}=C_{2}(hv-E_{g}\right)$$

**Figure 6.**
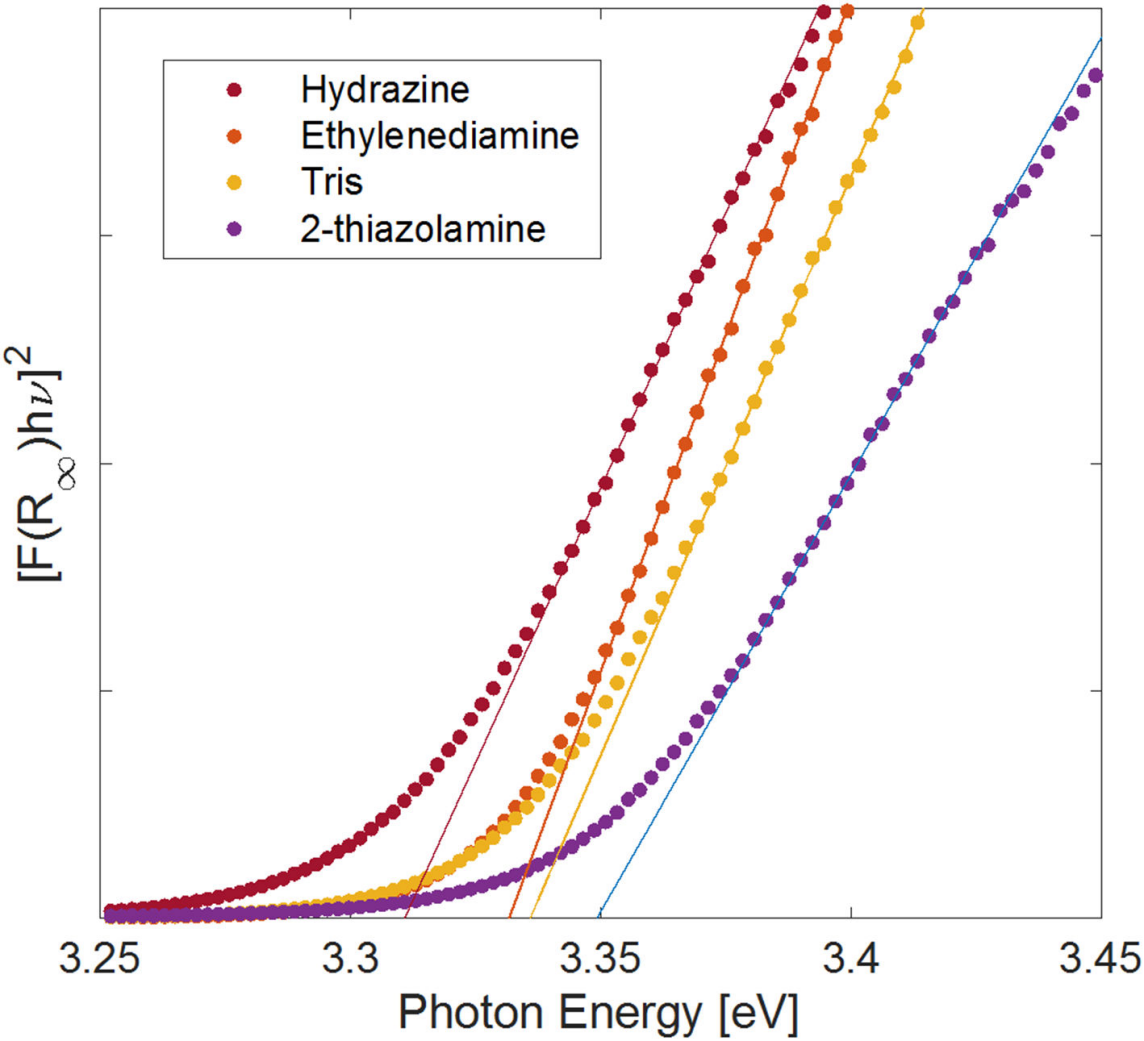
Kubelka–Munk transformed diffuse reflectance spectra of annealed ZnO powders prepared by rapid precipitation. The amine in the legend corresponds to the amine used during the synthesis of each material.

Optical bandgaps for the materials were found to be 3.31(1) eV for ZnO prepared using hydrazine, 3.33(1) eV for ZnO made with ethylenediamine, 3.34(2) eV for Tris, and 3.35(4) eV for ZnO made using 2-thiazolamine (Figure 6). These values are within three standard deviations of each other, compare well with what others have measured for ZnO nanoparticles [53,74], and are consistent with the 3.3 eV optical bandgap of bulk ZnO [76]. Changes in semiconductor bandgaps are generally attributed to reduction in crystallite size, the number of structural defects, and the overall crystallinity of the material [77,78]. Although the different amines can be used to modify the size and shape of ZnO particles and lead to different amounts of organic material attached to the nanoparticles, the crystallite size in the nanoparticles are very similar, as was observed with the Scherrer calculations, yielding similar bandgaps for all of the materials.

### 2.2. Photocatalytic Activity

Because of ZnO’s attractive physical and chemical properties, it has been extensively studied as a photocatalyst [79]. To explore how the synthesis conditions impact the material’s chemical reactivity, the ZnO prepared by rapid precipitation was used to photocatalyze the decolorization of the organic dye malachite green under ultraviolet (UVA) radiation. The mechanism for the photodegradation of dyes and other organic compounds by semiconductors has previously been described by many investigators [52,53,79-88]. Several catalysts have already been used to decolorize malachite green including, but not limited to, ZnO [53,81], NiS [82], TiO_2_ [83], Ag/TiO_2_/Nb_2_O_5_ composites [84], BiOBr composites [85], ZnO on activated carbon [86], Mn-doped BiOCl [87], and a Pd/WO_3_ mixture [88]. Under UV illumination in aqueous environments, ZnO generates reactive oxygen species responsible for the reactions that decolorize and degrade the dye [89-91]. In the reaction mechanism, ZnO absorbs a photon of UV light and generates an electron-hole pair on the particle surface (Equation (3)), with the electron being promoted to the conduction band (e_cb_^−^) and leaving the hole in the valence band (h_vb_^+^). The electrons react with surface adsorbed oxygen to form the superoxide radical ion O_2_^•−^ (Equation (4)). The holes react with adsorbed water molecules to form hydroxide radicals (HO^•^) and protons (Equation (5)), or in basic solutions, the holes can react with hydroxide molecules to produce additional hydroxide radicals (Equation (6)). The O_2_^•−^ can react with protons to form HO_2_^•^ (Equation (7)), which can further react to form hydrogen peroxide and O_2_ (Equation (8)). The peroxide then decomposes to generate more hydroxide radicals (Equation (9)). The hydroxide radicals generated by the mechanism are responsible for degrading the dye and converting the organic molecules to colorless intermediate products and, theoretically, carbon dioxide and water (Equation (10)). Saad et al. have proposed a degradation pathway for malachite green [44], and Yong and co-workers have identified 40 different intermediate products using liquid chromatography–mass spectrometry to analyze malachite green dye solutions during photodegradation [91].


(3) $$\text{ZnO}\;+hv\to e_{{\text{cb}}^{-}}+h_{{\text{vb}}^{+}}$$

(4) $$O_{2}+e_{{\text{cb}}^{-}}\to O_{2^{•-}}$$

(5) $$h^{+}+H_{2}O\to {\text{HO}}^{•}+H^{+}$$

(6) $$h^{+}+{\text{HO}}^{-}\to {\text{HO}}^{•}$$

(7) $${O_{2}}^{•-}+H^{+}\to {{\text{HO}}^{•}}_{2}$$

(8) $$2\;{{\text{HO}}^{•}}_{2}\to H_{2}O_{2}+O_{2}$$

(9) $$H_{2}O_{2}\to 2{\text{HO}}^{•}$$

(10) $${\text{HO}}^{•}+\text{dye}\to \text{intermediates}\to {\text{CO}}_{2}+H_{2}O$$

The decolorization of the dye proceeds by way of pseudo first-order rate kinetics. The reaction can be studied by measuring the dye’s absorbance (A) in solution over time (Equation (11)). The observed rate constants (*k*_obs_) include the degradation, and any physical adsorption of the dye onto the catalyst. The rate equation can be rearranged to give the rate constant as a positive slope (Equation (12)). In the equations, the initial absorbance of the dye in solution is A*.


(11) $$\text{ln}\;A=\text{−}k_{\text{obs}}t+\text{ln}\;A^{*}$$

(12) $$\text{ln}(A^{*}∕A)=k_{\text{obs}}t$$

**Figure 7.**
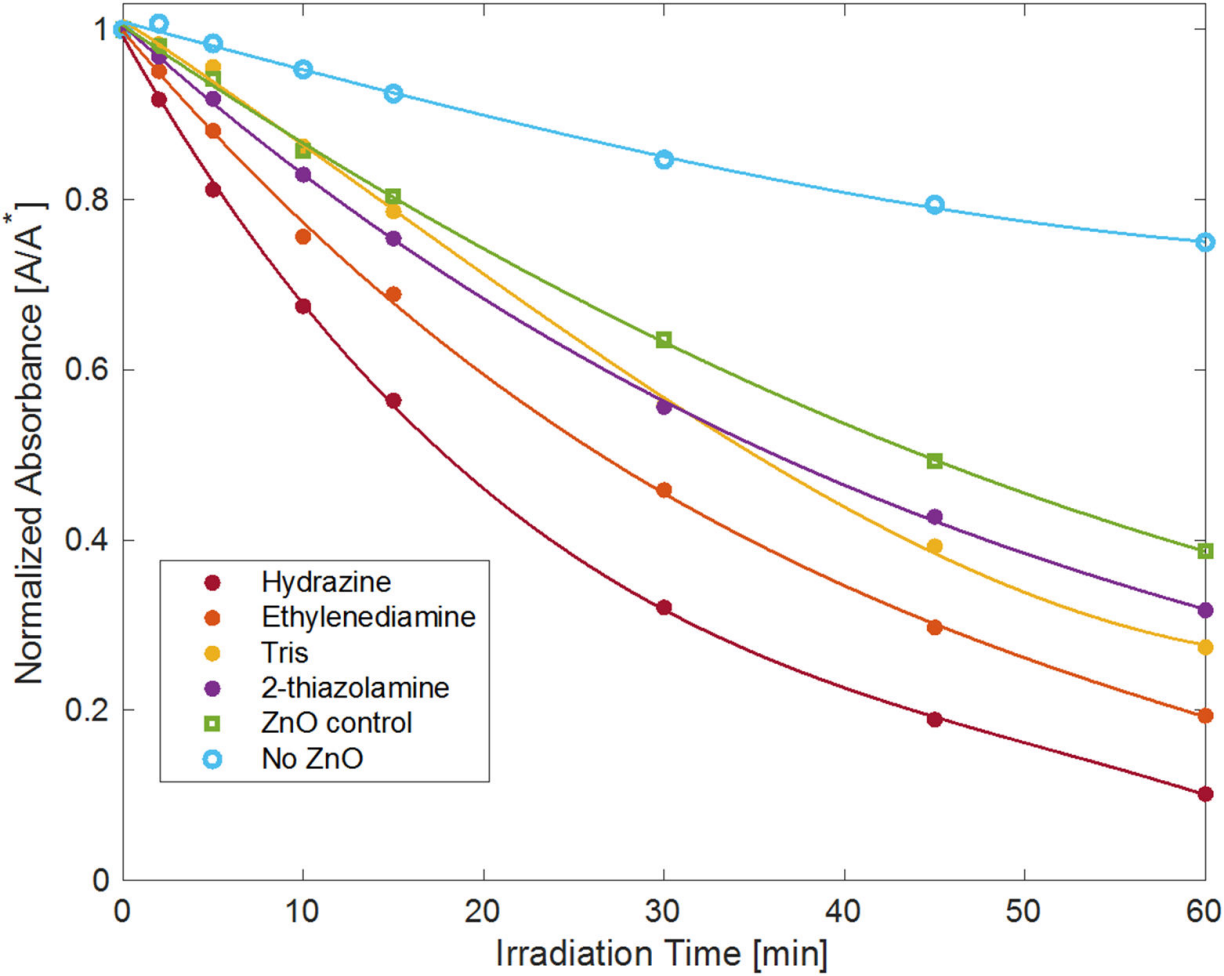
Absorption data for the decolorization of malachite green solutions using different zinc oxide photocatalysts prepared by rapid precipitation. The amines used to make the [Zn(acetate)_2_(amine)_2_] precursors are identified by the labels in the legend. The ZnO control was prepared using Zn(NO_3_)_2_ without added amines. “No ZnO” corresponds to the decolorization of malachite green with no catalyst in solution under UVA illumination.

**Figure 8.**
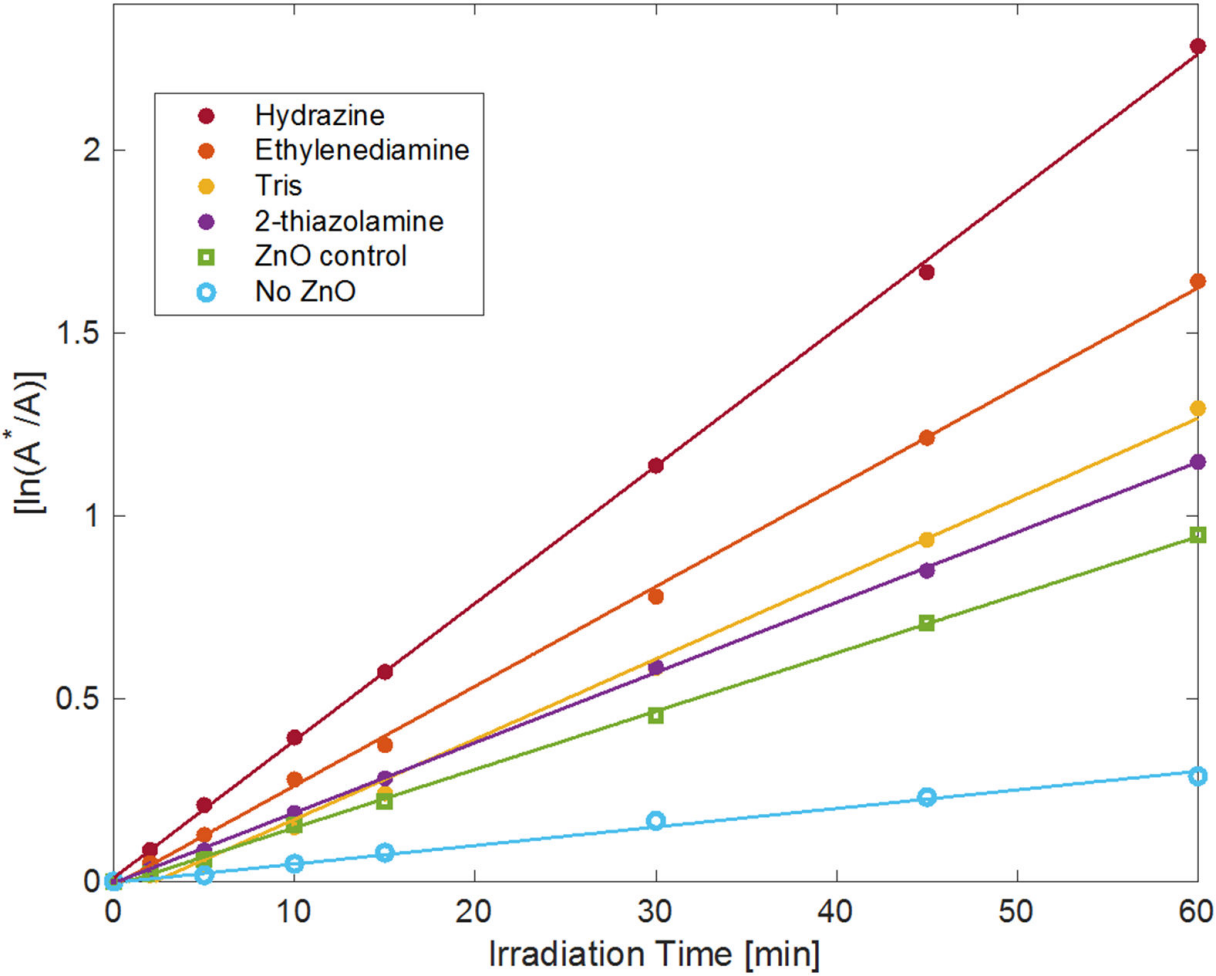
Absorption data of malachite green solutions during photodegradation by ZnO prepared by rapid precipitation. The amines used to make the [Zn(acetate)_2_(amine)_2_] precursors are identified by the labels in the legend. R^2^ values for all of the best fit lines exceed 0.997.

This work found that different sizes and morphologies of the ZnO particles influence the material’s ability to catalyze the photochemical reaction. In general smaller particles yielded faster decomposition rates than larger particles, given their larger surface area for particle sizes on the order of hundreds of nanometers to several micrometers. Additionally, rod and needle morphologies, with high surface area-to-volume ratios, also appear to increase photocatalytic effectiveness [92]. Wang et al. observed that 50 nm ZnO particles degraded methyl orange with the highest efficiency under UV light [93], and others observed that ZnO nanoparticles smaller than 50 nm were faster at degrading methylene blue than particles larger than 50 nm [49]. For this study, when ZnO particle size decreases to that produced by the rapid synthesis, the observed first-order rate constant (*k*_obs_) for the photodecomposition appears to be driven by multiple factors, including the quantity of ligands associated with the nanoparticles, as well as particle size and surface area (Figures 7 and 8 and Table 2). Zinc oxide prepared using hydrazine had the fastest rate constant, *k*_obs_ = 0.038(6) min^−1^, and degraded the dye more than twice as fast as the ZnO prepared using 2-thiazolamine, with *k*_obs_ = 0.019(2) min^−1^, even though the particles have similar size and surface area. ZnO prepared using ethylenediamine, *k*_obs_ = 0.024(1) min^−1^, and Tris, *k*_obs_ = 0.021(1) min^−1^, yielded intermediate decolorization rates, even though the material made using ethylenediamine had the largest particles and smallest surface area, whereas the material made using Tris had the smallest particles and largest surface area. A control was also prepared using Zn(NO_3_)_2_ with no added amine and used to decolor the dye solution and had a *k*_obs_ = 0.015(1) min^−1^. Additionally, it was observed that in the absence of a catalyst, the dye degrades under the UVA light with a *k*_obs_ = 0.005(2) min^−1^. The rate constants measured for the catalysts are consistent with what others have measured for the photodegradation of malachite green using ZnO [53-56,58,59]. Table 2 compares the photodegradation results for malachite green using zinc oxide prepared from several precursors.

The measurements were also obtained in the dark to determine the contribution of absorption on the decolorization constants. The *k*_obs,dark_ values for absorption in the dark were nearly identical for all of the materials, with the *k*_obs,dark_ = 0.007(1) min^−1^ for ZnO prepared from hydrazine, ethylenediamine, and 2-thizolamine and 0.008(1) min^−1^ for the ZnO prepared using Tris. Absorption onto the catalyst accounts for approximately one-third of the observed decolorization rate for the materials prepared using ethylenediamine, Tris, and 2-thiazolamine, but less than 20% of the *k*_*obs*_ for the material prepared using hydrazine.

As noted above in the discussion of the TGA results, ZnO produced using hydrazine and ethylenediamine retained the lowest amount of organic material and liberated the largest quantities of CO_2_ at temperatures under 200 °C, indicating that their surfaces are more readily accessible for absorbing CO_2_ than the materials prepared using the less volatile amines. This result likely contributes to their improved catalysis with a surface readily available in solution for water and oxygen sorption, which is necessary for the catalyst mechanism described above.

Additionally, much of the weight loss during heating from the sample made with hydrazine was in the form of water, likely from the decomposition of hydroxides. In contrast, the other materials all liberate more CO_2_ from bound hydrocarbons, allowing the hydrazine-derived material to have a more accessible surface and contributing to it being the fastest catalyst. The Tris and 2-thiazolamine-derived materials have hydrocarbons bound to them which impedes the ability of oxygen and water to reach the ZnO surface, slowing the degradation mechanism and kinetics. Even though both Tris and 2-thiazolamine have higher measured surface areas than the material prepared using ethylenediamine, their rates for decolorization are slower. Although the Tris-made ZnO contains more organic material than the 2-thiazolamine-made material, the Tris-made ZnO produced faster decolorization kinetics. The Tris-made material released more CO_2_ at temperatures below 200 °C than the ZnO made using 2-thiazolamine during TGA-MS measurements, suggesting that the Tris-made ZnO has a more accessible surface to CO_2_ in the air and perhaps H_2_O and O_2_ in solution. The Tris-made ZnO also contained smaller particle sizes with a higher surface area than the ZnO prepared using 2-thiazolamine. The authors speculate that these attributes contribute to the ZnO made using Tris degrading the dye faster than the ZnO made using 2-thiazolamine.

### 2.3. Catalyst Reusability

**Figure 9.**
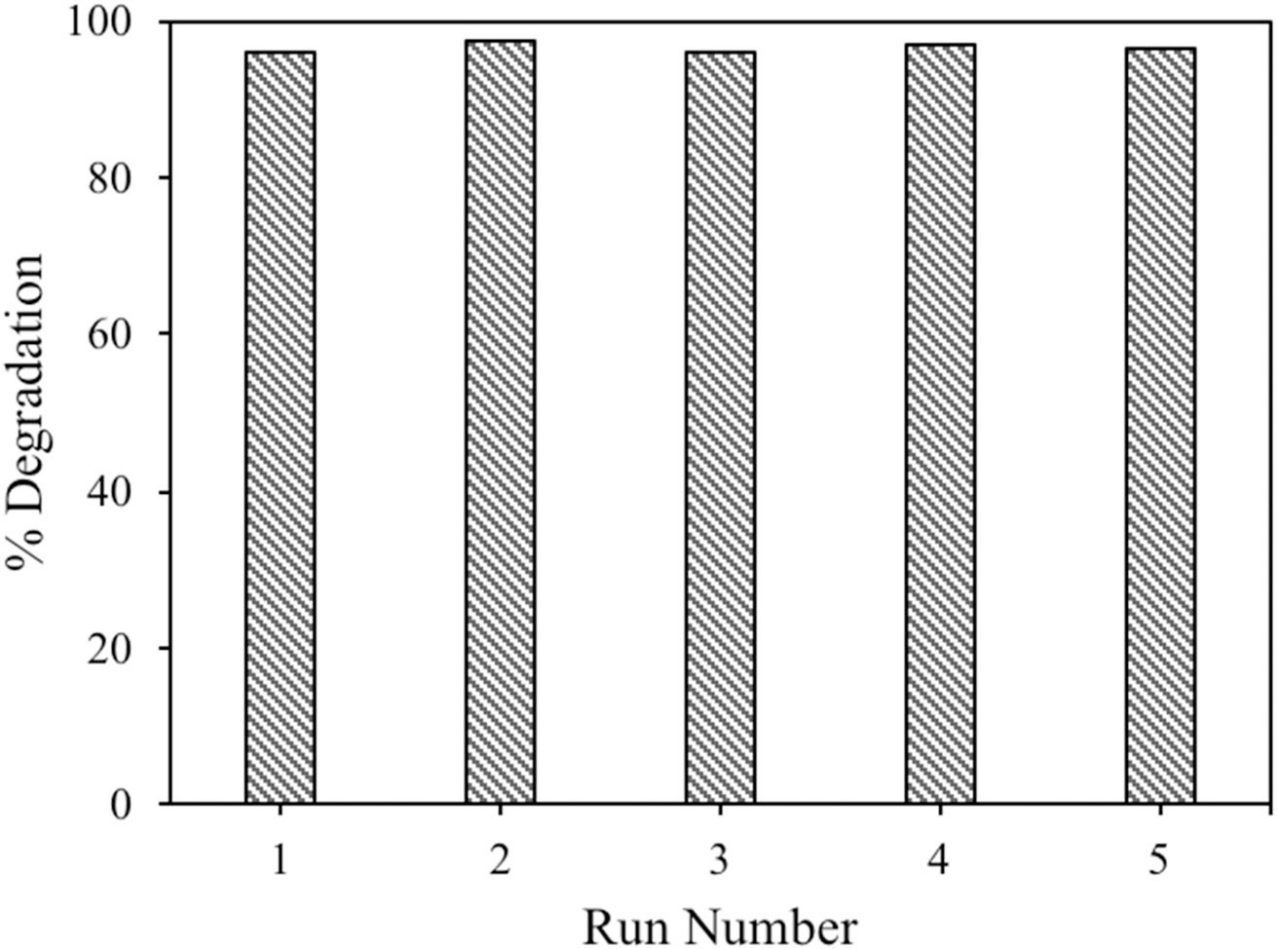
Reusability study for ZnO prepared using [Zn(acetate)_2_(hydrazine)_2_] under UVA illumination for two hours. A single sample of ZnO decolored five malachite green dye samples with minimal efficiency loss.

The ZnO made using hydrazine was evaluated for its reusability, as it had the highest rate constant in the study. A single sample of the material was used to decolorize five samples of the dye solution with no significant loss in catalytic efficiency (Figure 9). For these experiments, 0.100 g of catalyst was added to 50 mL of a 28.9 μM dye solution and allowed to stir for two hours under UVA illumination. At the end of each experiment, the nanomaterial was recovered by centrifugation and then added to the next dye solution. The percent dye decolorization ranged from 96–97% for all five samples, indicating very good recyclability of the material.

## 3. Materials and Methods

### 3.1. Techniques and Materials

Zinc acetate dihydrate (ACS reagent grade, Fisher Chemicals) was used as received. Amines were either used as received (ACS reagent grade, Sigma-Aldrich Chemicals) or distilled under nitrogen before use. All synthesis was done in containers open to the atmosphere. Solvents were either ACS or HPLC grade and used as received. Ultrapure water for solutions and synthesis was obtained using a Millipore Milli-Q system.

### 3.2. ZnO Precursor Isolation and Characterization

Each ZnO precursor was isolated from solution for characterization by ^1^H NMR and CHN elemental analysis to verify precursor composition. The synthesis and characterization of [Zn(acetate)_2_(Tris)_2_] and [Zn(acetate)_2_(ethylenediamine)] were reported in our previous publication [39].

For the synthesis of [Zn(acetate)_2_(2-thiazolamine)_2_], zinc acetate dihydrate (1.0311 g, 47 mmo) and 2-thiazolamine (0.9412 g, 94 mmol) were dissolved in a mixed solution of anhydrous ethanol (20 mL) and methanol (20 mL) under ambient conditions. The solution was stirred for one hour and then was evaporated to dryness under vacuum. Yield: 1.6863 g (93.5%). Soluble in dimethylsulfoxide, and methanol. Elem. Anal. for ZnC_10_H_14_N_4_O_4_S_2_: C = 31.30; H = 3.68; N = 14.60%. Found: C = 31.44; H = 3.65; N = 14.58%. ^1^H NMR (CD_3_OD): δ 2.015 (s, 6H, OOCC*H*_3_); 6.599 (s, 2H, NH_2_CSC*H*_2_CH_2_N), 6.943 (s, 2H, NH_2_CSCH_2_C*H*_2_N).

For the synthesis of [Zn(acetate)_2_(hydrazine)_2_], zinc acetate dihydrate (1.0314 g, 47 mmol) and hydrazine (0.5160 g) were dissolved in a mixed solution of anhydrous ethanol (20 mL) and water (20 mL) under ambient conditions. The solution was stirred for one hour and then was evaporated to dryness under vacuum. Yield: 0.9751 g (83.8%). Insoluble in all solvents. Elem. Anal. for ZnC_4_H_14_N_4_O_4_: C = 19.41; H = 5.70; N = 22.63%. Found: C = 19.37; H = 5.67; N = 22.52%.

### 3.3. ZnO Particle Synthesis

For a typical nanoparticle synthesis, zinc acetate dihydrate (5.3938 g, 24.6 mmol) was dissolved in 100 mL of methanol with stirring. An amine was then added (49.1 mmol for monodentate amines and 25.4 mmol for bidentate amines). The solution was allowed to stir for 30 min at room temperature, 100 mL of water was added, the solution was heated to 65 °C and rapidly titrated with 3 mL of an aqueous 50% NaOH solution to precipitate ZnO. Upon cooling, the reaction solutions were centrifuged to isolate the product, rinsed three times with water and three times with methanol, dried under vacuum, and then dried at 200 °C for two hours under a nitrogen atmosphere. The amines used in the synthesis included hydrazine, ethylenediamine, 2-thiazolamine, and tris(hydroxymethyl)aminomethane (Tris). The product prepared using hydrazine partially precipitates before the NaOH addition. Typical ZnO yield: 1.8089 g, 90%.

### 3.4. Powder X-Ray Diffraction

Powder X-ray diffraction (XRD) was utilized to confirm the crystallographic structure of the materials and estimate their particle size using the Scherrer equation [95]. All XRD measurements were performed on a Rigaku 2200 D/Max X-ray diffractometer. The diffractometer was equipped with a copper sealed tube anode utilizing Cu Kα radiation (λ = 1.5418 Å). Specimens were scanned from 20° to 95° 2Θ with a step size of 0.02° and a dwell time of 1 s. Phase composition was determined by comparison to the International Centre for Diffraction Data (ICDD) patterns.

### 3.5. Scanning Electron Microscopy

Field emission scanning electron microscopy (FESEM) (Hitachi, S-4500) was used to determine size and morphology of all ZnO samples. SEM micrographs were acquired and processed using Quartz PCI Version 8 image processing software. Accelerating voltages of 7 kV to 20 kV were used for imaging, with magnification ranging from 1000X to 200,000X, depending on particle size and morphology. The ZnO powders were deposited onto wet silver paint applied to the aluminum sample stage to minimize charging and loose particles in the FESEM vacuum chamber. The paint was allowed to dry, and then compressed air was applied to the sample to remove any loose particulates.

### 3.6. Transmission Electron Microscopy

Powders prepared by rapid addition of NaOH were characterized by Transmission Electron Microscopy (TEM). Sample preparation was done by dispersing 4 mg of the ZnO nanoparticles into 4 mL of methanol and ultrasonicating for five minutes. Immediately after ultrasonication, one drop of the ZnO nanoparticle suspension was placed on an ultrathin holey carbon grid (Ted Pella, product # 01824). Analysis of the ZnO nanoparticles was carried out using a Zeiss Libra 200 transmission electron microscope, and micrographs were acquired using a Gatan Orius CCD digital camera. Analytical TEM (ATEM) was employed by running scanning transmission electron microscopy (STEM) and performing X-ray energy dispersive spectroscopy (XEDS) to confirm the elemental composition of the nanoparticles. STEM images were acquired with a high angle annular dark field detector (HAADF). The Zeiss Libra 200 is equipped with a Noran XEDS system, with a Li drifted Si detector. All image acquisitions and XEDS measurements were carried out with an acceleration voltage of 200 kV and an emission current of 304 μA. XEDS measurements were made with a beam spot size of 20 nm. Average particle sizes and standard deviations were determined from measurements of 50–60 particles for each material.

### 3.7. Ultraviolet–Visible Diffuse Reflectance Spectroscopy

The optical band gap of the ZnO nanoparticles was determined at room temperature by diffuse reflectance, using an Agilent Cary 100 spectrophotometer equipped with an internal diffuse reflectance attachment and a powder sample cell. Samples were scanned from 300 to 500 nm. Data were transformed using the Kubelka–Munk equation [74,75]. The band gap was determined using the R software package grofit from the intersection of the tangent at the maximum slope of the smoothed cubic spline fit to the *x*-axis [96,97].

### 3.8. Surface Area Measurements

The surface area of the powders was measured with a surface area and pore size analyzer (Quantachrome Instruments, NOVA 2200 e, Boynton Beach, FL, USA). Approximately 0.3 gm of each powder was weighed in a bulbous glass vial, subsequently vacuum degassed at 200 °C for 12 h, and then backfilled with helium. Adsorption and desorption curves were then obtained at liquid-nitrogen temperatures using N_2_ as the adsorbate for P/Po ranging from 0.05 to 0.99. An automatic multipoint Brunauer, Emmett, and Teller (BET) model was fitted to the adsorption curve from P/Po ranging from 0.05 to 0.3 and was used to calculate the surface area. Before analyzing the ZnO samples, a certified standard (105.57 ± 4.41 m^2^/g) was measured to be 107.54 m^2^/g.

### 3.9. Thermal Analysis

All ZnO samples were analyzed by thermogravimetric analysis using a Mettler Toledo TGA/DSC1 thermal analyzer connected to a Pfieffer Vacuum ThermoStar mass spectrometer. Samples were heated with ramp rates ranging from 5–50 °C/min from 30 °C to 1000 °C in alumina crucibles in a dry air atmosphere. Blanks for each crucible were measured before each sample. Samples sizes of 10–20 mg were routinely used. Samples sizes of 50–60 mg were used when characterizing the volatile compounds produced during heating by mass spectrometry.

### 3.10. Photocatalytic Activity

The photocatalytic activity of the different ZnO nanoparticles was evaluated by photocatalytic decolorization of malachite green oxalate dye solutions (28.9 μM) under ultraviolet (UV) illumination. For these experiments, ZnO nanoparticles (0.1 g) were magnetically stirred in 10 mL of water for 10 min to disperse the nanoparticles in solution, then 50 mL of the dye solution was added and irradiated with UVA illumination (two 13W Damar 26289A F13TT/BLB bulbs). The intensity of the light at the surface of the dye was measured as approximately 3.4 W/m^2^ using a Vernier UVA Sensor. Aliquots of the dye solution were removed at specific time intervals (0, 2, 5, 10, 15, 30, 45, and 60 min), centrifuged for one minute at 12,000 rpm to isolate the ZnO, and the absorbance of the solution was measured at 617 nm in quartz cuvettes using an Agilent Cary 100 spectrophotometer. Experiments in the dark were performed in the absence of both UVA and ambient light. For consistency, all experiments were performed in the same 600 mL beaker using the same two-inch Teflon-coated stir bar to stir the solutions. Average kinetic rate constants with standard deviations were determined from seven to ten experiments using three different sample preparations with the same amine. All data were manipulated using Microsoft Excel.

## 4. Conclusions

We have demonstrated that [Zn(acetate)_2_(amine)_x_] compounds can be used to prepare wurtzite zinc oxide nanoparticles by alkali precipitation. The size and aspect ratio (length to width) of ZnO particles can be modified using different amines to produce needle-, rod- or plate-shaped particles. Nucleation kinetics can further control particle size, with larger particles prepared using slow precipitation rates and higher solution temperatures and fast precipitation rates and lower solution temperatures producing nanoparticles. Amines that resulted in the smallest aspect ratios during higher temperatures and slow precipitation rates yielded the smallest particles during rapid precipitation, and amines that yielded particles with large aspect ratios during slow precipitation produced the largest nanoparticles during rapid precipitation, even though all produced mostly spherical, hexagonal particles during rapid precipitation. The surface area of the nanomaterials was found to depend only on particle size, with the material with the smallest particles yielding the highest surface area. The optical bandgap of the materials was not altered by particle size or the amines bound to the precursor compounds, which is consistent with the materials having similar crystallite sizes, regardless of particle size. The different amines on the precursors alter the number of organic ligands associated with each material. In general, ZnO made using precursors containing less-volatile amines retain more organic material than those prepared using more-volatile amines. The presence of the organic material on the nanoparticles impacts the photocatalytic activity of the materials, with the ZnO containing the lowest amount of associated hydrocarbons yielding the highest rate constants for the decolorization of malachite green, regardless of particle size. [Zn(acetate)_2_(hydrazine)_2_] produced zinc oxide with the least organic material bound to the nanoparticles and degraded the dye the fastest. In recycling experiments, zinc oxide made using [Zn(acetate)_2_(hydrazine)_2_] degraded five malachite green solutions with minimal loss of efficiency.

## Supplementary Material

Figure S1 Figure S1: Absorption, desorption isotherms for BET surface area measurements for ZnO prepared using Tris.

Figure S2 Figure S2: Absorption, desorption isotherms for BET surface area measurements for ZnO prepared using 2-thiazolamine.

Figure S3 Figure S3: Absorption, desorption isotherms for BET surface area measurements for ZnO prepared using hydrazine.

Figure S4 Figure S4: Absorption, desorption isotherms for BET surface area measurements for ZnO prepared using ethylenediamine.

Figure S5 Figure S5: Thermogravimetric analysis (TGA) and mass spectrometry (MS) curves for the heating of ZnO prepared using [Zn(acetate)_2_(ethylenediamine)].

Figure S6 Figure S6: Thermogravimetric analysis (TGA) and mass spectrometry (MS) curves for the heating of ZnO prepared using [Zn(acetate)_2_(hydrazine)_2_].

Figure S7 Figure S7: Thermogravimetric analysis (TGA) and mass spectrometry (MS) curves for the heating of ZnO prepared using [Zn(acetate)_2_(2-thiazolamine)_2_].

Figure S8 Figure S8: Thermogravimetric analysis (TGA) and mass spectrometry (MS) curves for the heating of ZnO prepared using [Zn(acetate)_2_(Tris)_2_].

Figure S9 Figure S9: Thermogravimetric analysis (TGA) and mass spectrometry (MS) curves for the heating of ZnO prepared using Zn(acetate)_2_ with no added amine.

Figure S10 Figure S10: Thermogravimetric analysis (TGA) and mass spectrometry (MS) curves for the heating of ZnO prepared using Zn(nitrate)_2_ and ethylenediamine.

Figure S11 Figure S11: Thermogravimetric analysis (TGA) and mass spectrometry (MS) curves for the heating of ZnO prepared using Zn(nitrate)_2_ and 2-thiazolamine.

Figure S12 Figure S12: Thermogravimetric analysis (TGA) and mass spectrometry (MS) curves for the heating of ZnO prepared using Zn(nitrate)_2_ and hydrazine.

Figure S13 Figure S13: Thermogravimetric analysis (TGA) and mass spectrometry (MS) curves for the heating of ZnO prepared using Zn(nitrate)_2_ and Tris.

Figure S14 Figure S14: Infrared spectra of ZnO prepared using [Zn(acetate)_2_(Tris)_2_], [Zn(acetate)_2_(2-thiazolamine)_2_], [Zn(acetate)_2_(hydrazine)_2_] and [Zn(acetate)_2_(ethylenediamine)].

## Figures and Tables

**Table 1.** Size and surface area of the ZnO nanoparticles produced using the different [Zn(acetate)_2_(amine)_x_] precursors during rapid precipitation in methanol/water solutions. Sizes are average sizes measured with the TEM, and calculated with the Scherrer and surface area equations. Standard deviations for measurements are provided in parentheses.

Amine	Particle Size (nm) (TEM)	Particle Size (nm) (Scherrer)	Particle Size (nm) (Surface Area)	Surface Area (m^2^/g)
Tris	20(4)	15(1)	24.5	43.7
Hydrazine	27(7)	17(4)	30.0	35.6
2-Thiazolamine	34(9)	19(1)	30.5	35.1
Ethylenediamine	45(7)	18(4)	66.4	16.1

**Table 2.** Photodegradation of malachite green using zinc oxide made from different precursors.

Precursor	Dye Conc. (g/L)	Catalyst Conc. (g/L)	*k*_obs_ (min^−1^)	Ref
ZnCl_2_	0.01	0.2	0.010	[58]
Zn(acetate)_2_	0.2	0.2	0.014	[53]
Zn(acetate)_2_ + NH_3_	0.02	0.05	0.009	[94]
Zn(acetate)_2_ + TEA	0.01	0.12	0.0046	[56]
Zn(NO_3_)_2_ + PEG	0.2	0.2	0.023	[53]
Zn(NO_3_)_2_ + EDTA	.0093	0.2	0.058	[55]
[Zn(acetate)_2_(hydrazine)_2_]	0.027	1.67	0.038	this work
[Zn(acetate)_2_(ethylenediamine)]	0.027	1.67	0.024	this work
[Zn(acetate)_2_(Tris)_2_]	0.027	1.67	0.021	this work
[Zn(acetate)_2_(2-thiazolamine)_2_]	0.027	1.67	0.019	this work

TEA = triethylamine, PEG = polyethylene glycol, EDTA = ethylenediaminetetraacetic acid.

## References

[1] Janotti A; van de Walle CG; Fundamentals of zinc oxide as a semiconductor. Rep. Prog. Phys 2009, 72, 126501.

[2] Özgür Ü; Alivov YI; Liu C; Teke A; Reshchikov MA; Doğan S; Avrutin V; Cho S-J; Morko H A comprehensive review of ZnO materials and devices. J. Appl. Phys 2005, 98, 041301.

[3] Zang Z; Tang X; Enhanced fluorescence imaging performance of hydrophobic colloidal ZnO nanoparticles by a facile method. J. Alloys Compd 2015, 619, 98–101.

[4] Liu S; Yu B; Zhang H; Fei T; Zhang T Enhancing NO_2_ gas sensing performances at room temperature based on reduced graphene oxide-ZnO nanoparticles hybrids. Sens. Actuators B Chem 2014, 202, 272–278.

[5] Padmavathy N; Vijayaraghavan R Enhanced bioactivity of ZnO nanoparticles—An antimicrobial study. Sci. Technol. Adv. Mater 2008, 9, 035004. 27878001 10.1088/1468-6996/9/3/035004 PMC5099658

[6] Premanathan M; Karthikeyan K; Jeyasubramanian K; Manivannan G Selective toxicity of ZnO nanoparticles toward Gram-positive bacteria and cancer cells by apoptosis through lipid peroxidation. Nanomed. Nanotechnol. Biol. Med 2011, 7, 184–192. 10.1016/j.nano.2010.10.001. 21034861

[7] Babitha N; Priya LS; Christy SR; Manikandan A; Dinesh A; Durka M; Arunadevi S Enhanced Antibacterial Activity and Photo-Catalytic Properties of ZnO Nanoparticles: *Pedalium Murex* Plant Extract-Assisted Synthesis. J. Nanosci. Nanotechnol 2019, 19, 2888–2894. 10.1166/jnn.2019.16023. 30501796

[8] Zhou H; Li Z Synthesis of nanowires, nanorods and nanoparticles of ZnO through modulating the ratio of water to methanol by using a mild and simple solution method. Mater. Chem. Phys 2005, 89, 326–331. 10.1016/j.matchemphys.2004.09.006.

[9] Bhushan B; Murty B; Mondal K A new approach for synthesis of ZnO nanorod flowerets and subsequent pure free-standing ZnO nanorods. Adv. Powder Technol 2019, 30, 30–41. 10.1016/j.apt.2018.10.004.

[10] Cossuet T; Roussel H; Chauveau J-M; Chaix-Pluchery O; Thomassin J-L; Appert E; Consonni V Well-ordered ZnO nanowires with controllable inclination on semipolar ZnO surfaces by chemical bath deposition. Nanotechnology 2018, 29, 475601. 10.1088/1361-6528/aadf62. 30251706

[11] Liu B; Zeng HC Direct growth of enclosed ZnO nanotubes. Nano Res. 2009, 2, 201–209. 10.1007/s12274-009-9018-7.

[12] Choi K-S; Chang S-P Effect of structure morphologies on hydrogen gas sensing by ZnO nanotubes. Mater. Lett 2018, 230, 48–52. 10.1016/j.matlet.2018.07.031.

[13] Mina S-K; Manea RS; Joob O-S; Ganesha T; Choc BW; Han S-H; Upright-standing ZnO nano-sheets growth using wet chemistry. Curr. Appl. Phys 2009, 9, 492–495.

[14] Wang M; Luo Q; Hussain S; Liu G; Qiao G; Kim EJ Sharply-precipitated spherical assembly of ZnO nanosheets for low temperature H2S gas sensing performances. Mater. Sci. Semicond. Process 2019, 100, 283–289. 10.1016/j.mssp.2019.05.020.

[15] Li Y; Liu C-S Hydro/solvo-thermal synthesis of ZnO crystallite with particular morphology. Trans. Nonferrous Metal Soc. China 2009, 19, 399–403. 10.1016/s1003-6326(08)60285-x.

[16] Li P; Zhu S; Hu H; Guo L; He T Influence of defects in porous ZnO nanoplates on CO2 photoreduction. Catal. Today 2019, 335, 300–305. 10.1016/j.cattod.2018.11.068.

[17] Zhang L; Jeem M; Okamoto K; Watanabe S Photochemistry and the role of light during the submerged photosynthesis of zinc oxide nanorods. Sci. Rep 2018, 8, 177. 10.1038/s41598-017-18572-8. 29317711 PMC5760726

[18] Xu Z; Ben Y; Chen Z; Qi F Facile synthesis of snowflake-like ZnO nanostructures at low temperature and their super catalytic activity for the ozone decomposition. Mater. Res. Bull 2013, 48, 1725–1727. 10.1016/j.materresbull.2012.11.095.

[19] Sigoli FA; Davolos MR; Jafelicci M Jr. Morphological evolution of zinc oxide originating from zinc hydroxide carbonate. J. Alloys Compd 1997, 262–263, 292–295.

[20] Wang L; Muhammed M Synthesis of zinc oxide nanoparticles with controlled morphology. J. Mater. Chem 1999, 9, 2871–2878. 10.1039/a907098b.

[21] Liu Y; Liu Z; Wang G Synthesis and characterization of ZnO nanorods. J. Cryst. Growth 2003, 252, 213–218. 10.1016/s0022-0248(02)02518-6.

[22] Meagley KL; Garcia SP Chemical Control of Crystal Growth with Multidentate Carboxylate Ligands: Effect of Ligand Denticity on Zinc Oxide Crystal Shape. Cryst. Growth Des 2012, 12, 707–713. 10.1021/cg200992z.

[23] Anders CB; Eixenberger JE; Franco NA; Hermann RJ; Rainey KD; Chess JJ; Punnoose A; Wingett DG ZnO nano-particle preparation route influences surface reactivity, dissolution and cytotoxicity. Environ. Sci. Nano 2018, 5, 572–588. 29479436 10.1039/C7EN00888K PMC5823520

[24] Marin O; González V; Tirado M; Comedi D Effects of methanol on morphology and photoluminescence in solvothermal grown ZnO powders and ZnO on Si. Mater. Lett 2019, 251, 41–44.

[25] Andrés-Vergés M; Martínez-Gallego M Spherical and rod-like zinc oxide microcrystals: Morphological characterization and microstructural evolution with temperature. J. Mater. Sci 1992, 27, 3756–3762.

[26] Noack V; Eychmüller A Annealing of Nanometer-Sized Zinc Oxide Particles. Chem. Mater 2002, 14, 1411–1417. 10.1021/cm011262i.

[27] Ohyama M; Kouzuka H; Yoko T Sol-Gel preparation of ZnO films with extremely preferred orientation along (002) plane from zinc acetate solution. Thin Solid Films 1997, 306, 78–85.

[28] Yang Y; Chen H; Zhao B; Bao X Size control of ZnO nanoparticles via thermal decomposition of zinc acetate coated on organic additives. J. Cryst. Growth 2004, 263, 447–453. 10.1016/j.jcrysgro.2003.12.010.

[29] Zhang Z; Lu M; Xu H; Chin W-S Shape-Controlled Synthesis of Zinc Oxide: A Simple Method for the Preparation of Metal Oxide Nanocrystals in Non-aqueous Medium. Chem. Eur. J 2006, 13, 632–638. 10.1002/chem.200600293. 16991178

[30] Bacaksiz E; Parlak M; Tomakin M; Özçelik A; Karakız M; Altunbaş M The effects of zinc nitrate, zinc acetate and zinc chloride precursors on investigation of structural and optical properties of ZnO thin films. J. Alloy. Compd 2008, 466, 447–450. 10.1016/j.jallcom.2007.11.061.

[31] Ebrahimifard R; Abdizadeh H; Golobostanfard MR Controlling the extremely preferred orientation texturing of sol–gel derived ZnO thin films with sol and heat treatment parameters. J. Sol-Gel Sci. Technol 2020, 93, 28–35. 10.1007/s10971-019-05157-2.

[32] Ravichandran K; Begum NJ; Snega S; Sakthivel B Properties of Sprayed Aluminum-Doped Zinc Oxide Films—A Review. Mater. Manuf. Process 2016, 31, 1411–1423. 10.1080/10426914.2014.930961.

[33] Eensalu JS; Krunks M; Gromyko I; Katerski A; Mere A A comparative study on physical properties of Al-doped zinc oxide thin films deposited from zinc acetate and zinc acetylacetonate by spray pyrolysis. Energetika 2017, 63, 46–55 10.6001/energetika.v63i2.3519.

[34] Lanje AS; Sharma SJ; Ningthoujam RS; Ahn J-S; Pode RB; Low temperature dielectric studies of zinc oxide (ZnO) na-noparticles prepared by precipitation method. Adv. Powder Technol 2013, 24, 331–335.

[35] Li P; Wei Y; Liu H; Wang X-K Growth of well-defined ZnO microparticles with additives from aqueous solution. J. Solid State Chem 2005, 178, 855–860. 10.1016/j.jssc.2004.11.020.

[36] Cao Y; Miao L; Tanemura S; Tanemura M; Kuno Y; Hayashi Y; Low resistivity p-ZnO films fabricated by sol-gel spin coating. Appl. Phys. Lett 2006, 88, 251116.

[37] Kittilstved KR; Norberg NS; Gamelin DR; Chemical manipulation of high-TC ferromagnetism in ZnO diluted magnetic semiconductors. Phys. Rev. Lett 2005, 94, 147209. 15904107 10.1103/PhysRevLett.94.147209

[38] Vajargah PH; Abdizadeh H; Ebrahimifard R; Golobostanfard MR Sol-gel derived ZnO thin films: Effect of ami-no-additives. Appl. Surf. Sci. B 2013, 285, 732–743.

[39] Hyslop JS; Boydstun AR; Fereday TR; Rusch JR; Strunk JL; Wall CT; Pena CC; McKibben NL; Harris JD; Thurber A; Synthesis and characterization of [Zn(acetate)2(amine) ] compounds (x = 1 or 2) and their use as precursors to ZnO. Mater. Sci. Semicond. Process 2015, 38, 278–289. 10.1016/j.mssp.2015.04.001. 26085801 PMC4465802

[40] Culp SJ; Beland FA Malachite Green: A Toxicological Review. J. Am. Coll. Toxicol 1996, 15, 219–238. 10.3109/10915819609008715.

[41] Srivastava S; Sinha R; Roy D Toxicological effects of malachite green. Aquat. Toxicol 2004, 66, 319–329. 10.1016/j.aquatox.2003.09.008. 15129773

[42] Lee KM; Lai CW; Ngai KS; Juan JC Recent developments of zinc oxide based photocatalyst in water treatment technology: A review. Water Res. 2016, 88, 428–448. 10.1016/j.watres.2015.09.045. 26519627

[43] Oliveira AG; Andrade JDL; Montanha MC; Lima SM; Andrade LHDC; Hechenleitner AAW; Pineda EAG; de Oliveira DMF Decontamination and disinfection of wastewater by photocatalysis under UV/visible light using nano-catalysts based on Ca-doped ZnO. J. Environ. Manag 2019, 240, 485–493. 10.1016/j.jenvman.2019.03.124. 30965176

[44] Saad AM; Abukhadra MR; Ahmed SA-K; Elzanaty AM; Mady AH; Betiha MA; Shim J-J; Rabie AM Photocatalytic degradation of malachite green dye using chitosan supported ZnO and Ce–ZnO nano-flowers under visible light. J. Environ. Manag 2020, 258, 110043. 10.1016/j.jenvman.2019.110043. 31929075

[45] Tayyebi A; outokesh M; Tayebi M; Shafikhani A; Şengör SS ZnO quantum dots-graphene composites: Formation mecha-nism and enhanced photocatalytic activity for degradation of methyl orange dye. J. Alloys Compd 2016, 663, 738–749.

[46] Tayyebi A; Soltani T; Lee B-K; Outokesh M; Tayebi M Novel Visible Light Photocatalytic and Photoelectrochemical (PEC) Activity of Carbon-doped Zinc Oxide/Reduced Graphene Oxide: Supercritical Methanol Synthesis with Enhanced Photocor-rosion Suppression. J. Alloys Compd 2017, 723, 1001–1010.

[47] Tayebi M; Tayyebi A; Masoumi Z; Lee B-K Photocorrosion suppression and photoelectrochemical (PEC) enhancement of ZnO via hybridization with graphene nanosheets. Appl. Surf. Sci 2020, 502, 144189. 10.1016/j.apsusc.2019.144189.

[48] Elias; Uddin N; Saha J; Hossain A; Sarker D; Akter S; Siddiquey I; Uddin J A Highly Efficient and Stable Photocatalyst; N-Doped ZnO/CNT Composite Thin Film Synthesized via Simple Sol-Gel Drop Coating Method. Molecules 2021, 26, 1470. 10.3390/molecules26051470. 33800455 PMC7962968

[49] Shen W; Li Z; Wang H; Liu Y; Guo Q; Zhang Y Photocatalytic degradation for methylene blue using zinc oxide prepared by codeposition and sol-gel methods. J. Hazard. Mater 2008, 152, 172–175. 10.1016/j.jhazmat.2007.06.082. 17689008

[50] Shifu C; Wei Z; Sujuan Z; Wei L Preparation, characterization and photocatalytic activity of N-containing ZnO powder. Chem. Eng. J 2009, 148, 263–269. 10.1016/j.cej.2008.08.039.

[51] Yang Y; Li Y; Zhu L; He H; Hu L; Huang J; Hu F; He B; Ye Z Shape control of colloidal Mn doped ZnO nanocrystals and their visible light photocatalytic properties. Nanoscale 2013, 5, 10461–10471. 10.1039/c3nr03160h. 24056701

[52] Giahi M; Farajpour G; Taghavi H; Shokri S Preparation of Photocatalytic ZnO Nanoparticles and Application in Photo-chemical Degradation of Betamethasone Sodium Phosphate Using Taguchi Approach. Russ. J. Phys. Chem. A 2014, 88, 1241–1247.

[53] Saikia L; Bhuyan D; Saikia M; Malakar B; Dutta DK; Sengupta P Photocatalytic performance of ZnO nanomaterials for self sensitized degradation of malachite green dye under solar light. Appl. Catal. A Gen 2015, 490, 42–49. 10.1016/j.apcata.2014.10.053.

[54] Khezami L; Taha KK; Ghiloufi I; Mir LE Adsorption and photocatalytic degradation of malachite green by vanadium doped zinc oxide nanoparticles. Water Sci. Technol 2015, 73, 881–889. 10.2166/wst.2015.555. 26901732

[55] Meena S; Vaya D; Das BK Photocatalytic degradation of Malachite Green dye by modified ZnO nanomaterial. Bull. Mater. Sci 2016, 39, 1735–1743. 10.1007/s12034-016-1318-4.

[56] Kaneva N; Bojinova A; Papazova K Photocatalytic degradation of Reactive Black 5 and Malachite Green with ZnO and lanthanum doped nanoparticles. J. Phys. Conf. Ser 2016, 682, 12022. 10.1088/1742-6596/682/1/012022.

[57] Horzum N; Hilal ME; Isık T Enhanced bactericidal and photocatalytic activities of ZnO nanostructures by changing the cooling route. New J. Chem 2018, 42, 11831–11838. 10.1039/c8nj01849a.

[58] Babajani N; Jamshidi S Investigation of photocatalytic malachite green degradation by iridium doped zinc oxide nanoparti-cles: Application of response surface methodology. J. Alloys Compd 2019, 782, 533–544.

[59] Rabie AM; Abukhadra MR; Rady AM; Ahmed SA; Labena A; Mohamed HSH; Betiha MA; Shim J-J Instantaneous photocatalytic degradation of malachite green dye under visible light using novel green Co–ZnO/algae composites. Res. Chem. Intermed 2020, 46, 1955–1973. 10.1007/s11164-019-04074-x.

[60] Vitiello G; Iervolino G; Imparato C; Rea I; Borbone F; de Stefano L; Aronne A; Vaiano V F-doped ZnO nano- and meso-crystals with enhanced photocatalytic activity in diclofenac degradation. Sci. Total Environ 2021, 762, 143066. 10.1016/j.scitotenv.2020.143066. 33127133

[61] Moezzi A; Cortie M; McDonagh A Transformation of zinc hydroxide chloride monohydrate to crystalline zinc oxide. Dalton Trans. 2016, 45, 7385–7390. 10.1039/c5dt04864h. 27030646

[62] Dutta D Optimization of process parameters and its effect on particle size and morphology of ZnO nanoparticle synthesized by sol–gel method. J. Sol-Gel Sci. Technol 2016, 77, 48–56. 10.1007/s10971-015-3827-9.

[63] Harun K; Hussain F; Purwanto A; Sahraoui B; Zawadzka A; Mohamad AA Sol–gel synthesized ZnO for optoelectronics applications: A characterization review. Mater. Res. Express 2017, 4, 122001. 10.1088/2053-1591/aa9e82.

[64] Spanhel L Colloidal ZnO nanostructures and functional coatings: A survey. J. Sol-Gel Sci. Technol 2006, 39, 7–24. 10.1007/s10971-006-7302-5.

[65] Briois V; Giorgetti C; Baudelet F; Blanchandin S; Tokumoto MS; Pulcinelli SH; Santilli CV Dynamical Study of ZnO Nanocrystal and Zn-HDS Layered Basic Zinc Acetate Formation from Sol−Gel Route. J. Phys. Chem. C 2007, 111, 3253–3258. 10.1021/jp0662909.

[66] Zheng Z; Butynska R; Serrano CV; Marty J-D; Mingotaud C; Kahn ML One-Step Synthesis of Hybrid Liquid-Crystal ZnO Nanoparticles: Existence of a Critical Temperature Associated with the Anisotropy of the Nanoparticles. Chem. Eur. J 2016, 22, 15614–15618. 10.1002/chem.201602742. 27599122

[67] Jia G; Xu S; Wang A Emerging strategies for the synthesis of monodisperse colloidal semiconductor quantum rods. J. Mater. Chem. C 2015, 3, 8284–8293. 10.1039/c5tc01234a.

[68] Herrera-Rivera R; de la Olvera ML; Maldonado A Synthesis of ZnO Nanopowders by the Homogeneous Precipitation Method: Use of Taguchi’s Method for Analyzing the Effect of Different Variables. J. Nanomater 2017, 2017, 4595384. 10.1155/2017/4595384.

[69] Alnarabiji MS; Yahya N; Hamed Y; Ardakani SEM; Azizi K; Klemeš JJ; Abdullah B; Tasfy SFH; Hamid SBA; Nashed O Scalable bio-friendly method for production of homogeneous metal oxide nanoparticles using green bovine skin gel-atin. J. Cleaner Prod 2017, 162, 186–194.

[70] Li J-G; Ikegami T; Wang Y; Mori T 10-mol%-Gd2O3-Doped CeO2 Solid Solutions via Carbonate Coprecipitation: A Com-parative Study. J. Am. Ceram. Soc 2003, 86, 915–921.

[71] Kanaparthi S; Singh SG Chemiresistive Sensor Based on Zinc Oxide Nanoflakes for CO2 Detection. ACS Appl. Nano Mater 2019, 2, 700–706. 10.1021/acsanm.8b01763.

[72] Quy CT; Thai NX; Hoa ND; Le DTT; Hung CM; Duy NV; Hieu NV C2H5OH and NO2 sensing properties of ZnO nanostructures: Correlation between crystal size, defect level and sensing performance. RSC Adv. 2018, 8, 5629–5639. 35542445 10.1039/c7ra13702h PMC9078170

[73] Shaporev AS; Ivanov VK; Baranchikov AE; Polezhaeva OS; Tret’Yakov YD ZnO formation under hydrothermal conditions from zinc hydroxide compounds with various chemical histories. Russ. J. Inorg. Chem 2007, 52, 1811–1816. 10.1134/s0036023607120017.

[74] Morales AE; Mora ES; Pal U Use of diffuse reflectance spectroscopy for optical characterization of un-supported nanostructures. Rev. Mex. Fís. S 2007, 53, 18–22.

[75] Kubelka P; Munk F Ein Beitrag zur Optik der Farbanstriche. Z. Tech. Phys 1931, 12, 593–601.

[76] Srikant V; Clarke DR On the optical band gap of zinc oxide. J. Appl. Phys 1998, 83, 5447.

[77] Ramelan AH; Wahyuningsih S; Munawaroh H; Narayan R ZnO wide bandgap semiconductors preparation for optoe-lectronic devices. IOP Conf. Ser. Mater. Sci. Eng 2017, 176, 012008.

[78] Eixenberger JE; Anders CB; Wada K; Reddy KM; Brown RJ; Moreno-Ramirez J; Weltner AE; Karthik C; Tenne DA; Fologea D; Defect Engineering of ZnO Nanoparticles for Bioimaging Applications. ACS Appl. Mater. Interfaces 2019, 11, 24933–24944. 10.1021/acsami.9b01582. 31173687 PMC7010230

[79] Theerthagiri J; Salla S; Senthil RA; Nithyadharseni P; Madankumar A; Arunachalam P; Maiyalagan T; Kim H-S A review on ZnO nanostructured materials: Energy, environmental and biological applications. Nanotechnology 2019, 30, 392001. 10.1088/1361-6528/ab268a. 31158832

[80] Rauf MA; Ashraf SS Fundamental principles and application of heterogeneous photocatalytic degradation of dyes in solu-tion. Chem. Eng. J 2009, 151, 10–18.

[81] Rabieh S; Bagheri M; Heydari M; Badiei E Microwave assisted synthesis of ZnO nanoparticles in ionic liquid [Bmim]cl and their photocatalytic investigation. Mater. Sci. Semicond. Process 2014, 26, 244–250. 10.1016/j.mssp.2014.05.013.

[82] Prasad KS; Prajapati S; Selvaraj K Efficient sorption and photocatalytic degradation of malachite green dye onto NiS na-noparticles prepared using novel green approach. Korean J. Chem. Eng 2015, 32, 1986–1992.

[83] Jo W-K; Park GT; Tayade RJ Synergetic effect of adsorption on degradation of malachite green dye under blue LED irra-diation using spiral-shaped photocatalytic reactor. J. Chem. Technol. Biotechnol 2015, 90, 2280–2289.

[84] Goswami T; Reddy KM; Bheemaraju A Silver Nanocluster Anchored TiO2/Nb2O5 Hybrid Nanocomposite as Highly Efficient and Selective Visible-Light Sensitive Photocatalyst. ChemistrySelect 2019, 4, 6790–6799.

[85] Nethaji S; Tamilarasan G; Neehar P; Sivasamy A Visible light photocatalytic activities of BiOBr-activated carbon (derived from waste polyurethane) composites by hydrothermal process. J. Environ. Chem. Eng 2018, 6, 3735–3744.

[86] Ghaedi M; Ansari A; Habibi MH; Asghari AR Removal of malachite green from aqueous solution by zinc oxide nanopar-ticle loaded on activated carbon: Kinetics and isotherm study. J. Ind. Eng. Chem 2014, 20, 17–28.

[87] Pare B; Sarwan B; Jonnalagadda SB Photocatalytic mineralization study of malachite green on the surface of Mn-doped BiOCl activated by visible light under ambient condition. Appl. Surf. Sci 2011, 258, 247–253. 10.1016/j.apsusc.2011.08.040.

[88] Liu Y; Ohko Y; Zhang R; Yang Y; Zhang Z Degradation of malachite green on Pd/WO3 photocatalysts under simulated solar light. J. Hazard. Mater 2010, 184, 386–391. 10.1016/j.jhazmat.2010.08.047. 20855152

[89] Qi K; Cheng B; Yu J; Ho W Review on the improvement of the photocatalytic and antibacterial activities of ZnO. J. Alloy. Compd 2017, 727, 792–820. 10.1016/j.jallcom.2017.08.142.

[90] Ancona A; Dumontel B; Garino N; Demarco B; Chatzitheodoridou D; Fazzini W; Engelke H; Cauda V Lipid-Coated Zinc Oxide Nanoparticles as Innovative ROS-Generators for Photodynamic Therapy in Cancer Cells. Nanomaterials 2018, 8, 143. 10.3390/nano8030143. PMC5869634 29498676

[91] Yong L; Zhanqi G; Yuefei J; Xiaobin H; Cheng S; Shaogui Y; Lianhong W; Qingeng W; Die F Photodegradation of mal-achite green under simulated and natural irradiation: Kinetics, products, and pathways. J. Hazard. Mater 2015, 285, 127–136. 25497025 10.1016/j.jhazmat.2014.11.041

[92] Hepp AF; Bailey SG; McNatt JS; Chandrashekhar MVS; Harris JD; Rusch AW; Nogales KA; Goettsche KV; Hanson W; Amos D; Novel Materials, Processing and Device Technologies for Space Exploration with Potential Dual-Use Applications. In NASA Technical Memorandum; NASA/TM—2015-218866; National Aeronautics and Space Administration: Brook park, OH, USA, 2015.

[93] Wang H; Xie C; Zhang W; Cai S; Yang Z; Gui Y Comparison of dye degradation efficiency using ZnO powders with various size scales. J. Hazard. Mater 2007, 141, 645–652. 10.1016/j.jhazmat.2006.07.021. 16930825

[94] Sharma S; Mehta SK; Kansal SK N doped ZnO/C-dots nanoflowers as visible light driven photocatalyst for the degradation of malachite green dye in aqueous phase. J. Alloys Compd 2017, 699, 323–333.

[95] Langford JI; Wilson AJC Scherrer after sixty years: A survey and some new results in the determination of crystallite size. J. Appl. Crystallogr 1978, 11, 102–113. 10.1107/s0021889878012844.

[96] Team R. Core. R: A Language and Environment for Statistical Computing. The R Foundation for Statistical Computing. 2013, URL http://www.R-project.org/.

[97] Kahm M; Hasenbrink G; Lichtenberg-Fraté H; Ludwig J; Kschischo M grofit: Fitting Biological Growth Curves with R. J. Stat. Softw 2010, 33, 1–21. 10.18637/jss.v033.i07.20808728

