# Supplementary material for: Zinc–Acetate–Amine Complexes as Precursors to ZnO and the Effect of the Amine on Nanoparticle Morphology, Size, and Photocatalytic Activity
Source: Catalysts. Author manuscript; Available in PMC 2022 Nov 18. (PMC9673400; doi:10.3390/catal12101099)
Supplement: Figure S3 — Absorption, desorption isotherms for BET surface area measurements for ZnO prepared using hydrazine. [file NIHMS1846495-supplement-Figure_S3.pdf]

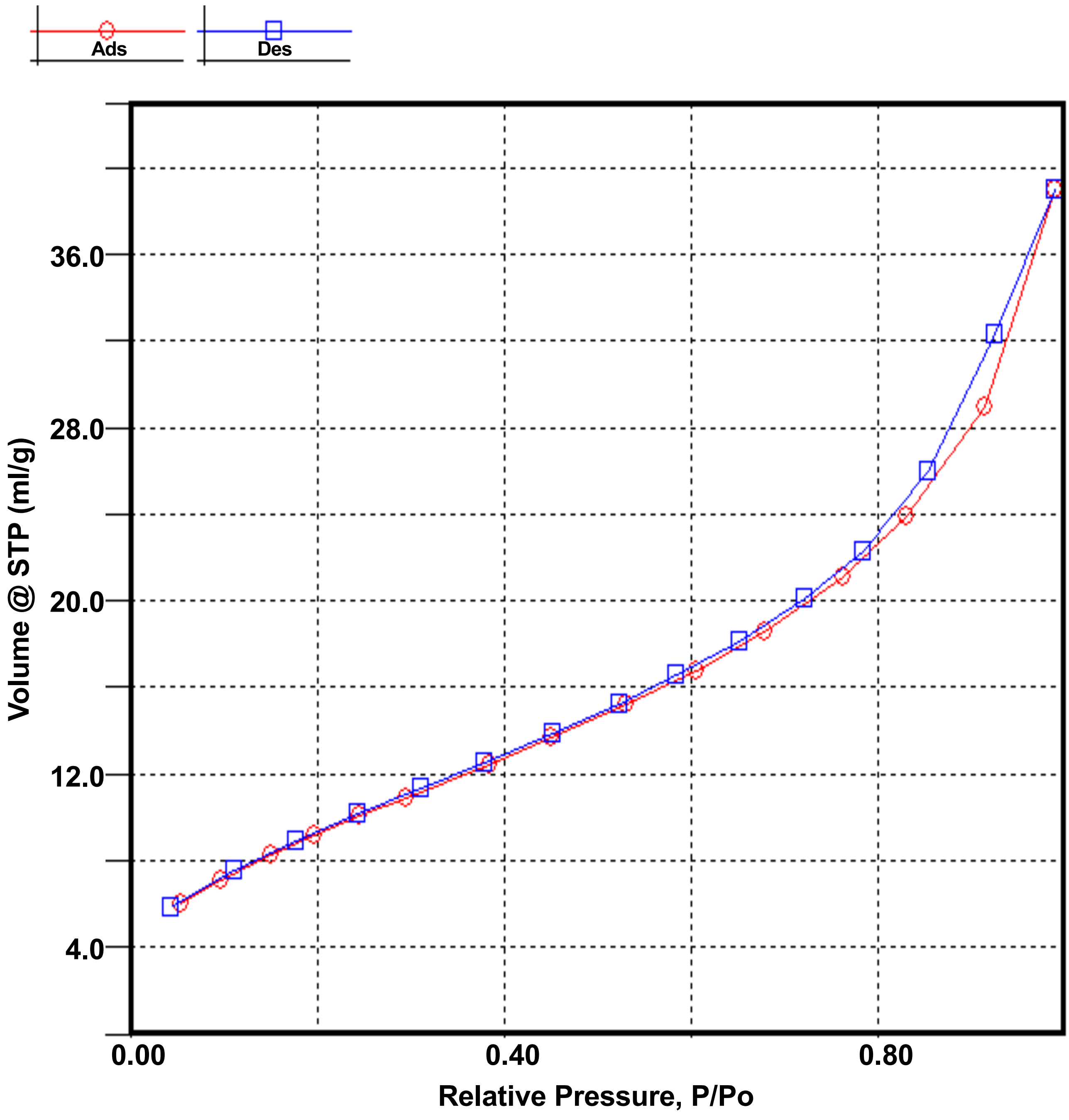

Figure S3. Absorption (red circles), desorption (blue squares) isotherms for BET surface area measurements for ZnO prepared using hydrazine.
