# Supplementary material for: Zinc–Acetate–Amine Complexes as Precursors to ZnO and the Effect of the Amine on Nanoparticle Morphology, Size, and Photocatalytic Activity
Source: Catalysts. Author manuscript; Available in PMC 2022 Nov 18. (PMC9673400; doi:10.3390/catal12101099)
Supplement: Figure S6 — Thermogravimetric analysis (TGA) and mass spectrometry (MS) curves for the heating of ZnO prepared using [Zn(acetate)2(hydrazine)2]. [file NIHMS1846495-supplement-Figure_S6.docx]

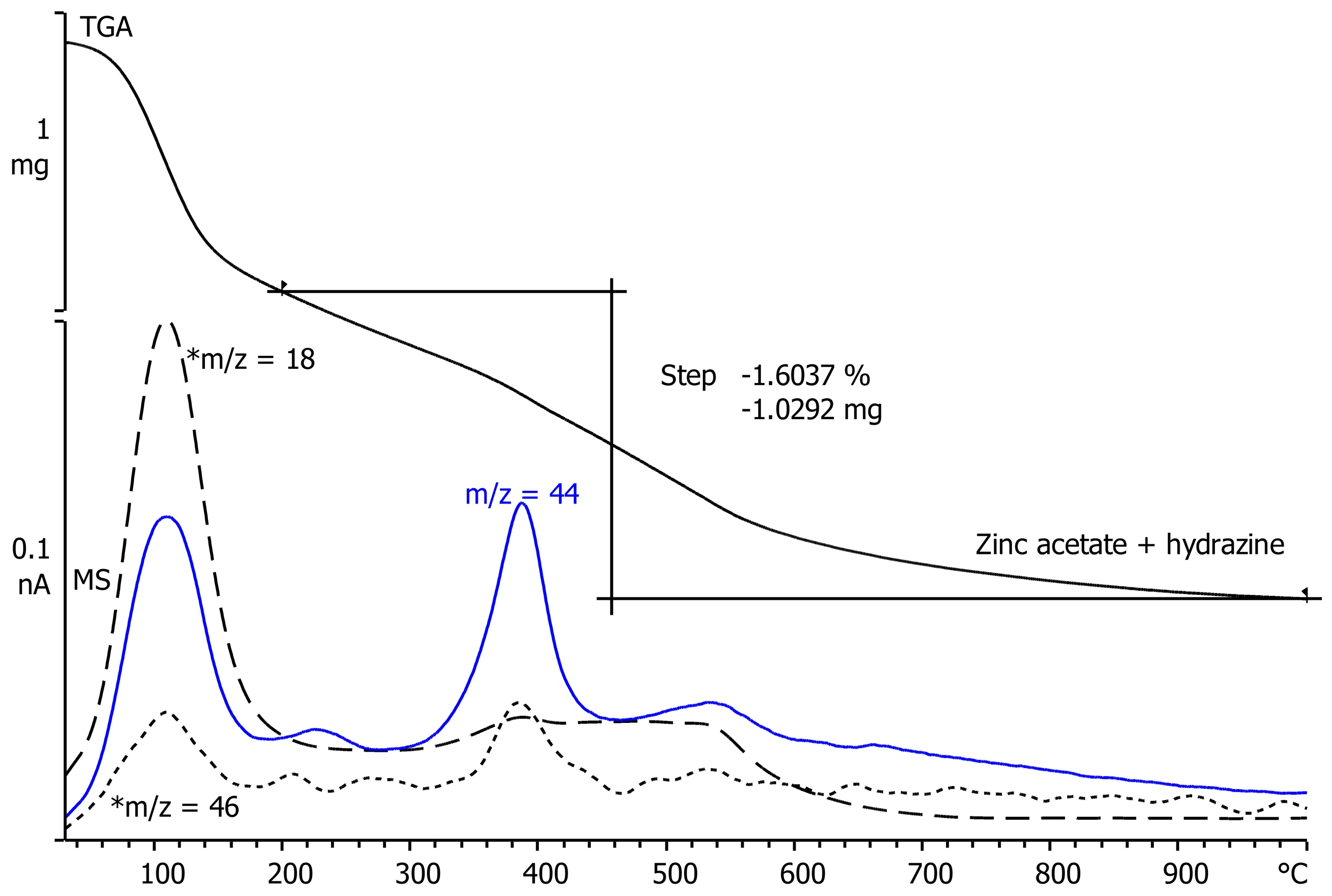


Figure S6. Thermogravimetric analysis (TGA) and mass spectrometry (MS) curves for the heating of ZnO prepared using [Zn(acetate)_2_(hydrazine)_2_] to 1000˚C in dry air at a rate of 20˚C/min. *The mass spectrograms for *m*/*z* = 18 and m/z = 46 are shown at 10% and 10,000%, respectively, to keep all mass signals on the same scale.
