# Supplementary material for: Zinc–Acetate–Amine Complexes as Precursors to ZnO and the Effect of the Amine on Nanoparticle Morphology, Size, and Photocatalytic Activity
Source: Catalysts. Author manuscript; Available in PMC 2022 Nov 18. (PMC9673400; doi:10.3390/catal12101099)
Supplement: Figure S9 — Thermogravimetric analysis (TGA) and mass spectrometry (MS) curves for the heating of ZnO prepared using Zn(acetate)2 with no added amine. [file NIHMS1846495-supplement-Figure_S9.docx]

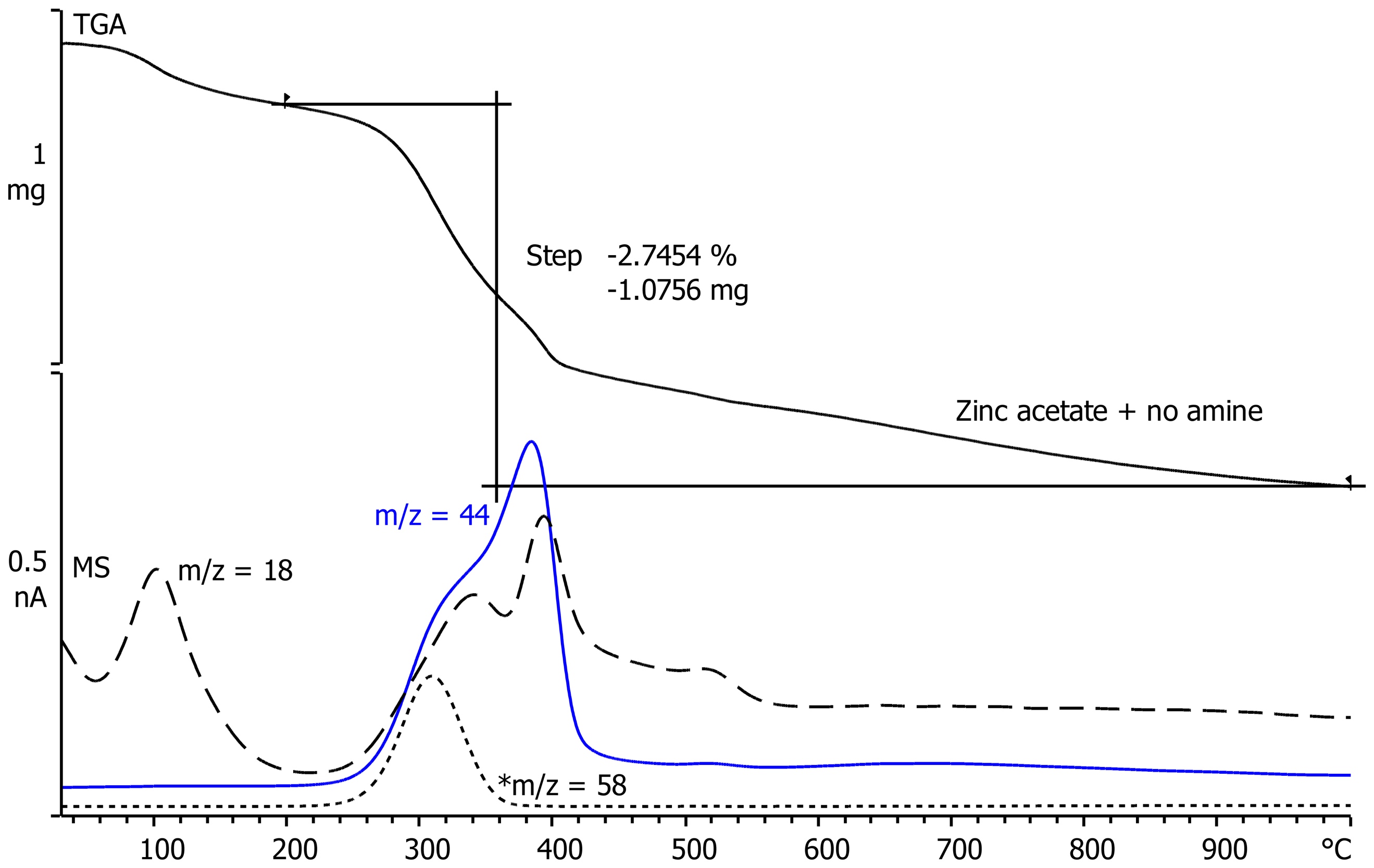


Figure S9. Thermogravimetric analysis (TGA) and mass spectrometry (MS) curves for the heating of ZnO prepared using Zn(acetate)_2_ with no added amine to 1000˚C in dry air at a rate of 20˚C/min. *The mass spectrogram for *m*/*z* = 58 is shown at 2,000% to keep all mass signals on the same scale.
