# Supplementary material for: Zinc–Acetate–Amine Complexes as Precursors to ZnO and the Effect of the Amine on Nanoparticle Morphology, Size, and Photocatalytic Activity
Source: Catalysts. Author manuscript; Available in PMC 2022 Nov 18. (PMC9673400; doi:10.3390/catal12101099)
Supplement: Figure S14 — Infrared spectra of ZnO prepared using [Zn(acetate)2(Tris)2], [Zn(acetate)2(2-thiazolamine)2], [Zn(acetate)2(hydrazine)2] and [Zn(acetate)2(ethylenediamine)]. [file NIHMS1846495-supplement-Figure_S14.docx]

Transmission (%, a.u.)

Wavenumber (cm^-1^)

Figure S14. IR spectra of ZnO prepared using [Zn(acetate)_2_(Tris)_2_], [Zn(acetate)_2_(2-thiazolamine)_2_], [Zn(acetate)_2_(hydrazine)_2_] and [Zn(acetate)_2_(ethylenediamine)].
